# Filling the gaps in icosahedral superatomic metal clusters

**DOI:** 10.1093/nsr/nwae174

**Published:** 2024-05-28

**Authors:** Wei-Miao He, Jia-Hua Hu, Yu-Jia Cui, Jing Li, Yu-Bing Si, Shuai-Bo Wang, Yu-Jing Zhao, Zhan Zhou, Lu-Fang Ma, Shuang-Quan Zang

**Affiliations:** College of Chemistry, Zhengzhou University, Zhengzhou 450001, China; College of Chemistry, Zhengzhou University, Zhengzhou 450001, China; College of Chemistry, Zhengzhou University, Zhengzhou 450001, China; College of Chemistry, Zhengzhou University, Zhengzhou 450001, China; School of Science, Xuchang University, Xuchang 461000, China; College of Chemistry, Zhengzhou University, Zhengzhou 450001, China; College of Chemistry, Zhengzhou University, Zhengzhou 450001, China; College of Chemistry, Zhengzhou University, Zhengzhou 450001, China; College of Chemistry, Zhengzhou University, Zhengzhou 450001, China; College of Chemistry and Chemical Engineering, Henan Key Laboratory of Function-Oriented Porous Materials, Luoyang Normal University, Luoyang 471934, China; College of Chemistry, Zhengzhou University, Zhengzhou 450001, China; College of Chemistry and Chemical Engineering, Henan Key Laboratory of Function-Oriented Porous Materials, Luoyang Normal University, Luoyang 471934, China; College of Chemistry, Zhengzhou University, Zhengzhou 450001, China

**Keywords:** icosahedral superatomic metal clusters, analog clusters, photoluminescence, alloying, near-infrared quantum yield

## Abstract

Chemically modified superatoms have emerged as promising candidates in the new periodic table, in which Au_13_ and its doped M*_n_*Au_13−_*_n_* have been widely studied. However, their important counterpart, Ag_13_ artificial element, has not yet been synthesized. In this work, we report the synthesis of **Ag_13_** nanoclusters using strong chelating ability and rigid ligands, that fills the gaps in the icosahedral superatomic metal clusters. After further doping **Ag_13_** template with different degrees of Au atoms, we gained insight into the evolution of their optical properties. Theoretical calculations show that the kernel metal doping can modulate the transition of the excited-state electronic structure, and the electron transfer process changes from local excitation (LE) to charge transfer (CT) to LE. This study not only enriches the families of artificial superatoms, but also contributes to the understanding of the electronic states of superatomic clusters.

## Teaser

The authors report the first icosahedral superatomic silver nanocluster (Ag_13_), and investigate the evolution of its optical properties and the differences in luminescence origin from Ag_13_ to Au_13_.

## INTRODUCTION

Atomically precise silver nanoclusters have been greatly enriched in terms of both numbers and properties [1–16], as well as the enormous progress in the field of structural growth evolution [17–21]. All these deepen our understanding of the property modulation of silver clusters. It is well known that the doping of metal atoms can effectively modulate the optical properties of nanoclusters by adjusting the energy level difference and changing the structural rigidity [22–26]. Bakr’s research group has demonstrated the construction of a pair of analog clusters (Au_25_ and Ag_25_), which is of great significance for understanding the evolution and properties of different metal nanoclusters [3]. Based on that, Jin's research group used Ag_25_, Au*_x_*Ag_25−_*_x_*, and Au_25_ as templates to study the change of the quantum yield (QY) induced by the change of electron vibration coupling via doping [27]. However, nanoclusters usually consist of a kernel and a motif, and the formation of the kernel structure is expected to play a critical role in the early stage of cluster growth. Therefore, capturing and understanding the kernel structure is indispensable in studying cluster growth and performance. On the other hand, the existence of peripheral motifs in silver nanoclusters blurs the research on the kernel structure, although they contribute to the stability of the clusters. This also introduces complex doping sites, impeding visualization of the effects of different metal doping on kernels [22–24,28,29]. Hence, the construction of novel gold and silver cluster analogs without motifs is required for working out the relationship between the kernel and other properties.

As classical magic-number clusters with closed-shell geometry, icosahedral M_13_-type clusters are considered to be thermodynamically stable [30–44] and thus are ideal candidates to be used for the construction of nanocluster analogs. Although Au_13_ has been widely studied in the fields of luminescence, chirality, and catalysis [29–34], the homologous Ag_13_ has not yet been reported. Interestingly, Ag_13_ is observed in the cores of many silver nanoclusters with core-shell structures [3,4,7,17,41–54], which indicates that Ag_13_ may be the embryonic state of such silver nanoclusters during their formation and evolution. Hence, capturing Ag_13_ helps to understand the growth process from simple silver complexes to complicated silver nanoclusters. Unfortunately, the icosahedron Ag_13_ has not been reported so far.

**Scheme 1.**
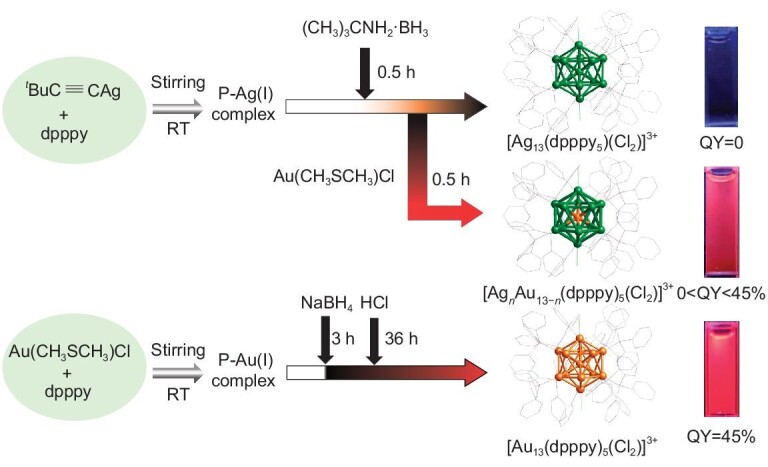
Synthetic routes for **Ag_13_, Au*_n_*Ag_13−_*_n_*** and **Au_13_**.

The clusters synthesized from 2,6-(diphenylphosphonyl) pyridine (dpppy) and transition metals are typically high in crystallinity and exhibit exceptional luminescent properties, since these two precursors present distinctive coordination modes, strong chelating ability, and robust structural rigidity [55–58]. Herein, we used dpppy as a protective ligand to construct a pair of 13 core icosahedral silver and gold nanoclusters with molecular formulas of [Ag_13_(dpppy)_5_(Cl)_2_](SbF_6_)_3_ (abbreviated as **Ag_13_**) and [Au_13_(dpppy)_5_(Cl)_2_](SbF_6_)_3_ (abbreviated as **Au_13_**) through different reduction strategies (Scheme 1). The prepared **Ag_13_** and **Au_13_** are identical in size, superatomic electronic structure, charge, and crystal structure. **Ag_13_** in the solution state was not photoluminescent in contrast with the strong near-infrared (NIR) luminescence observed in that of **Au_13_** with an ultrahigh quantum yield (QY = 45%) that is quite rare in gold nanoclusters [59–72]. A series of alloy nanoclusters (**Au*_n_*Ag_13−_*_n_***) were obtained by doping *in situ*, which exhibited significant gold content-dependent PL intensity, lifetime, and PLQY. More importantly, the uniform nano-micelles (**Au_13_@PLGA**) were obtained by encapsulating **Au_13_** with methoxypolyethylene glycol-poly(lactic-co-glycolic acid) (mPEG-PLGA), which presents great application potential in cell imaging.

## RESULTS AND DISCUSSION

### Structure of Ag_13_, Au_1_Ag_12_ and Au_13_

**Figure 1.**
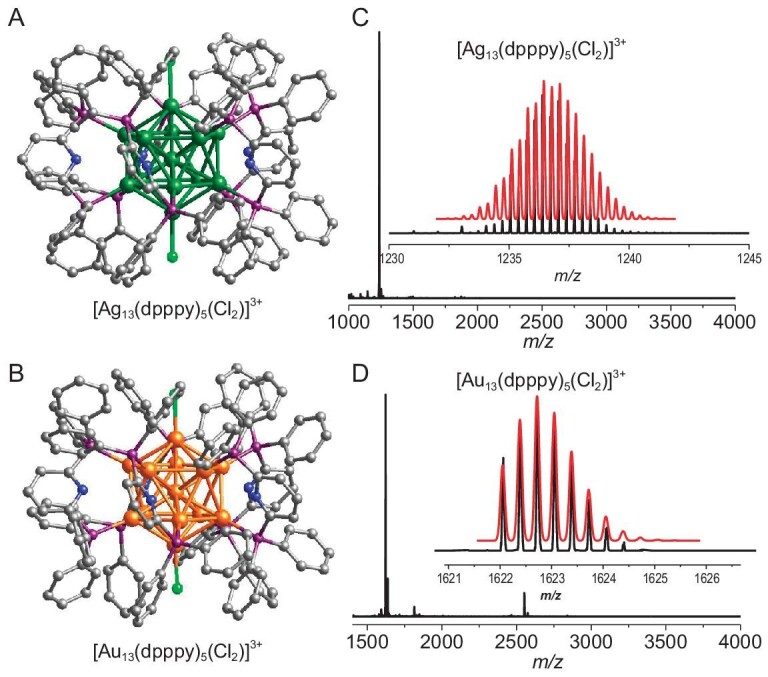
The structures of (A) **Ag_13_** and (B) **Au_13_** (omitting the hydrogen atom for clarity). The mass spectra of (C) **Ag_13_** and (D) **Au_13_**. The insets show the experimental (black trace) and simulated (red trace) isotopic patterns of the molecular ion peaks.

Single-crystal X-ray structure revealed **Ag_13_** crystallizing in the *P*-1 space group, and the cluster core was found to adopt an icosahedral configuration (Fig. 1A), in which the ten silver atoms at the waist are protected by five dpppy ligands while the silver atom at the top is capped by a chlorine atom. The icosahedral **Ag_13_** structure is composed of 20 triangular faces and a single Ag atom wrapped within. The average Ag···Ag distance between the central Ag atom and the Ag_12_ shell is 2.764 Å, which is shorter than that of the Ag_12_ shell (2.906 Å). **Au_13_** is an analog cluster of **Ag_13_**, and they are almost identical in terms of the metal atom number, ligands, superatomic electronic configuration, and atomic arrangement (Fig. 1B). Both of these two nanoclusters have abundant molecular interactions (Fig. S1).

**Figure 2.**
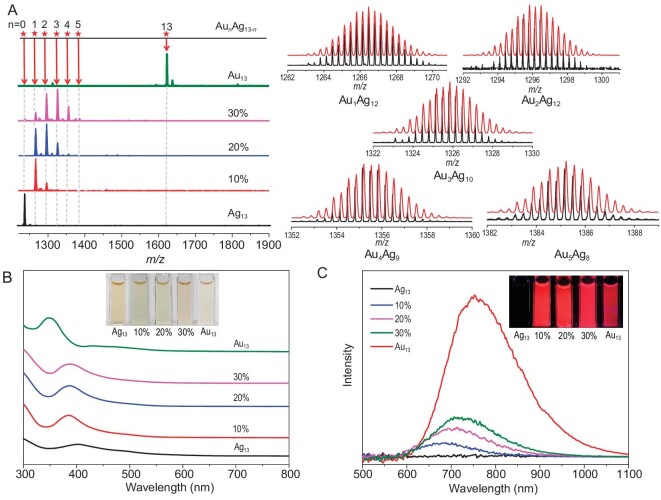
(A) Positive mode ESI-MS of **Ag_13_, Au*_n_*Ag_13−_*_n_*** (10%, 20% and 30%), and **Au_13_**_._ Inset: the experimental (black trace) and simulated (red trace) isotopic patterns of the molecular ion peaks. (B) UV/visible (Vis) and (C) PL spectra of **Ag_13_, Au*_n_*Ag_13−_*_n_*** (10%, 20% and 30%), and **Au_13_**. Insets: photographs of **Ag_13_, Au*_n_*Ag_13−_*_n_*** and **Au_13_** under ambient light and UV lamp (in DMSO, 33 ppm).

Electrospray ionization mass spectrometry (ESI-MS) was used to characterize the chemical composition and the charge state of **Ag_13_, Au_13_** and their alloy nanoclusters in solution. A clean signal was observed for **Ag_13_** and **Au_13_** at *m*/*z* = 1236.7 and 1622.7, respectively, which is consistent with the simulated data (Fig. 1C, D). Importantly, the ESI-MS curves of the alloy nanoclusters present a series of molecular ion peaks corresponding to the different numbers of gold atoms doped into the silver clusters. According to the mass between difference peaks, the number of doped gold atoms increased (from one to five) with the gold consumption, keeping the total number of metal atoms at 13 (Fig. 2A). Moreover, the results of inductively coupled plasma emission spectrometry (ICP) show that the Ag content of 10%, 20% and 30% (Au content) **Au*_n_*Ag_13−_*_n_*** gradually decreases, while Au content gradually increases (Table S1). Although the ICP results for 10% and 20% **Au*_n_*Ag_13−_*_n_*** show a small difference in Au content and are very close to that of **Au_1_Ag_12_**, these slight variations in gold content could result in significant differences in their optical properties, which is consistent with the reported literature [22–25]. The full X-ray photoelectron spectroscopy (XPS) spectra of **Ag_13_, Au_13_** and **Au*_n_*Ag_13−_*_n_*** revealed that the composition of the elements in the samples was consistent with the results obtained from single-crystal resolution (Fig. S2). As shown in Fig. S3A, B, high-resolution Ag 3d and Au 4f spectra of **Ag_13_** and **Au_13_** indicated that the presence of Ag^0^ in **Ag_13_** and **Au*_n_*Ag_13−_*_n_***, as well as the presence of Au^0^ in **Au_13_** and **Au*_n_*Ag_13−_*_n_***. In comparison to **Ag_13_**, the binding energy of **Au*_n_*Ag_13−_*_n_*** shifts towards higher binding energy values with increasing Au content (Fig. S3C), suggesting that Au doping can lead to subtle alterations in the electronic state of Ag. Similar to its effect on the binding energy of Ag, heteroatom doping also induce changes in the binding energy of Au (Fig. S3D). Due to the difference in ICP and XPS results of **Au*_n_*Ag_13−_*_n_***, we managed to obtain *n*-dependent structures by adjusting the gold doping ratio (Fig. S4). Unfortunately, only a structure with one Au atom replacing the Ag atom in the center of **Ag_13_**, namely **Au_1_Ag_12_**(Fig. S5) was harvested, which probably was due to its high thermodynamic stability similar to that of Au_1_Ag_24_ and Au_1_Ag_28_ [24,25]. Notably, we found that **Ag_13_, Au_1_Ag_12_** and **Au_13_** are chiral, but in the form of racemates (Figs S6–S8). Moreover, we tested the time-dependent ultraviolet (UV) absorption spectra of **Ag_13_** in different solvents at room temperature, and the results showed that its dimethyl sulfoxide (DMSO), dichloromethane (DCM), or acetone (AC) solution has attained certain stability in a short space of time (Fig. S9).

### Luminescence properties of Ag_13_, Au*_n_*Ag_13−_*_n_* and Au_13_

Although **Ag_13_** and **Au_13_** are analogs, their optical properties are quite different. From the absorption spectra, it is clear that the main peak of **Ag_13_** is located at 403 nm, while it has shifted to 386 nm after gold doping, and further blue shifted to 348 nm when **Ag_13_** becomes its analog **Au_13_**(Fig. 2B and Fig. S10). As for the luminescent property, **Ag_13_** exhibited red emission in its solid state, while almost no signal can be detected in its solution state (Fig. S11), which is reminiscent of the aggregation-induced emission (AIE) behavior. The luminescence of **Ag_13_** DMSO solution can be re-illuminated by adding deionized water (Fig. S12) or lowering the temperature to 83 K (Fig. S13), indicating that the interaction between the silver clusters and the solvent quenches their luminescence. After mixing **Ag_13_** with polymathic methacrylate (PMMA) to form a film (**Ag_13_**@PMMA), its emission also showed the intrinsic nature of **Ag_13_** in the solid state (Fig. S14). **Au_13_** displayed NIR luminescence in both solid and solution states with emission peaks located at 798 nm and 763 nm, respectively (Fig. S15). Excitingly, the QY of **Au_13_** was as high as 45% in the solution state, much higher than that in the solid state (20%, Table S2), which could be attributed to self-quenching as a result of intermolecular interactions in the solid state [73]. Although both **Ag_13_** and **Au_13_** have red luminescence and microsecond lifetimes in the solid state (Figs S16, S17), their solution luminescence properties are significantly different (Fig. S18). To further investigate the change of luminescence in solution during the evolution from **Ag_13_** to **Au_13_**, doping was used to construct a series of alloy nanoclusters. The emission center of alloy clusters gradually shifted from 680 nm to 720 nm with increasing gold content, and their intensity gradually increased and reached the maximum at 30% doping, which was still much weaker than that of **Au_13_** (Fig. 2C). Alloy clusters also showed bright luminescence in the solid state (Fig. S19), and their emission wavelength gradually red shifted from 645 to 700 nm with the increase in gold content (Fig. S20). Moreover, the luminescence of the solution was illuminated and gradually enhanced, and the photoluminescence lifetime and QY for **Au_13_** also gradually increased and reached 3.5 μs and 45%, respectively (Table S2 and Fig. S21). The microsecond lifetime implied inherent phosphorescence properties (**Ag_13_, Au_13_** and **Au*_n_*Ag_13−_*_n_***), which were further confirmed by their luminescence quenching both in solid and solution in the presence of oxygen (Figs S22–S25).

### Theoretical calculations of Ag_13_, Au*_n_*Ag_13−_*_n_* and Au_13_

To figure out the relationship between electronic structure and optical properties during the evolution, density functional theory (DFT) calculations were conducted for the different model clusters. All three clusters have eight ‘free’ valence electrons, leading to a superatomic configuration of 1S^2^|1P^6^ according to the Aufbau rule. By analyzing the DFT calculated results (Figs S26A–C, and S27–S29), it was found that the highest occupied molecular orbitals (HOMO, P*_x_* orbital) and HOMO-1 (P*_y_* orbital) are doubly degenerated in all of the three clusters, while the P*_z_* orbital (HOMO-2) has much lower energy than P*_x_* and P*_y_*, which might be due to the influence of the Cl p orbitals [31]. As shown in Fig. S26D–F, the UV spectra of the three clusters (**Ag_13_, Au_1_Ag_12_** and **Au_13_**) calculated by time-dependent DFT (TD-DFT) agree well with the measurements. According to the energy arrangement of the molecular orbitals (MOs) (Fig. S26G–I), the absorption of **Ag_13_** at 310 nm (a: HOMO-4→LUMO + 1, HOMO-1→LUMO + 31. LUMO stands for the lowest unoccupied molecular orbital.) is mainly produced by the combined effect of metal-metal electron transfer (MMCT) and metal-ligand electron transfer (MLCT), while the absorption at 403 nm (b: HOMO-2→ LUMO + 3) and the shoulder peaks at 485 nm (c: HOMO-1→LUMO + 1 and HOMO-1→LUMO + 2) are mainly due to MMCT. Compared to **Ag_13_**, the absorption of **Au_1_Ag_12_** in 386 nm is mainly attributed to ligand-metal electron transfer (LMCT, a: HOMO-2→LUMO + 7; b: HOMO-1→LUMO + 13; c: HOMO-1→LUMO + 11), while the absorption at 450 nm is mainly attributed to MMCT (d: HOMO-1→LUMO + 1, HOMO-1→LUMO + 2). The peak of **Au_13_** at 348 nm, is mainly attributed to electrons from HOMO-4→LUMO + 3, HOMO-3→LUMO + 4. The shoulder peak at 450 nm is mainly attributed to electrons jumping from HOMO-1→LUMO + 1, and HOMO-1→LUMO + 2. The results of the calculations demonstrated that, despite the similar structures of **Ag_13_, Au_1_Ag_12_** and **Au_13_**, their electron transfer pathways are distinct, which is the basis for their varied optical properties.

**Figure 3.**
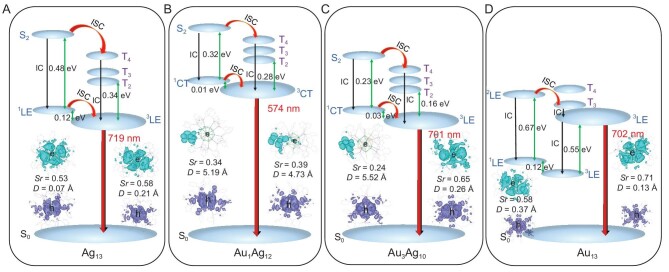
Energy diagrams of (A) **Ag_13_**, (B) **Au_1_Ag_12_**, (C) **Au_3_Ag_10_** and (D) **Au_13_**. Images of the hole and electron pairs. The *Sr* index is defined as the full space integration of a function (Sr(r)) describing the overlap between electron and hole distributions, and the *D* index is the distance between a hole and an electron center of mass.

To gain deeper insights into the impact of different core atomic compositions on the mechanism of the phosphorescence, we selected the two most classic **Ag_13_** and **Au_13_** clusters for excited state structure optimization. Simultaneously, we theoretically constructed alloy clusters **Au_1_Ag_12_** and **Au_3_Ag_10_**, with additional details provided in Figs S30 and S31. Based on the optimized S_1_ and T_1_/T_2_ excited state geometric structures of **Au*_n_*Ag_13−_*_n_*** (*n* = 0, 1, 3, 13), the fluorescence and phosphorescence radiative transition properties are studied. Fig. 3 shows the distribution of the hole and electron pairs during the S_1_→S_0_ and T_1_/T_2_→S_0_ vertical transitions for **Au*_n_*Ag_13−_*_n_*** (*n* = 0, 1, 3, 13) by natural transition orbital (NTO) analysis. As for **Ag_13_, Au_1_Ag_12_** and **Au_3_Ag_10_**, the calculated vertical emission energy of T_1_ to S_0_ agrees well with the experimental results (Fig. 3A–C and Table S3). For **Au_13_** (Fig. 3D), the calculated vertical emission energy of S_1_ to S_0_ is 1.35 eV (919 nm), and T_1_ to S_0_ is 1.27 eV (974 nm) which is much smaller than that of experimental results (1.57 eV, 798 nm). But the vertical emission energy of T_2_→S_0_ is 1.77 eV (702 nm), which is close to the experimental result (1.57 eV, 798 nm). The phosphorescent emission of **Au_13_** observed in the experiment appears to be mainly attributed to the T_2_→S_0_ transition. The generation of T_2_ phosphorescence can be explained by a large Δ*E*(T_2 _− T_1_) value (0.55 eV) and $\langle {{\mathrm{T}}_2}| {{\mathrm{\hat H}}soc} |{{\mathrm{S}}_0}\rangle $ (124.16 cm^−1^), causing T_2_ phosphorescence to compete favorably with internal conversion (IC). The large Δ*E*(S_2 _− S_1_) value (0.67 eV) leads to slow S_2_→S_1_ internal conversion, and strong spin-orbit coupling between the S_2_ and high-lying triplet states T*_m_* (T_4_, T_3_, T_2_) leads to fast S_2_→T*_m_* (Tables S4, S5). Therefore, the phosphorescence mechanism of **Au_13_** can be better understood from S_0_→S*_n_*→S_2_→T*_m_*→T_2_→S_0_.

The long lifetime (τ_p_) and high quantum efficiency (Φ_p_) conflict in principle [74]. It is thus desirable to gain a better understanding of the factors governing Φ_p_. The intersystem crossing (ISC) rate is mainly determined by the spin−orbit coupling (SOC) $\langle {\mathrm{S}}| {{\mathrm{\hat H}}soc} |{\mathrm{T}}\rangle $ and the energy difference Δ*E*_ST_ between S and T states. The ISC rate can be qualitatively estimated through El-Sayed's rules [75]. According to these rules, the ISC rate is relatively large if the transition between the S and T states involves a change of molecular orbital type (Tables S10–S13). In **Ag_13_** and **Au_13_**, both S_1_ and T_1_ display obvious local excitation (LE) features due to the small *D* values (*D* index <0.5 Å). Obviously, both the $\langle {{\mathrm{S}}_1}| {{\mathrm{\hat H}}soc} |{{\mathrm{T}}_1}\rangle $ and $\langle {{\mathrm{T}}_1}| {{\mathrm{\hat H}}soc} |{{\mathrm{S}}_0}\rangle $ are less than 17 cm^−1^ in **Ag_13_**, indicating a slow ISC process of S_1_→T_1_ and T_1_→S_0_ [70], thus leading to the low Φ_p_ (exp. 1.5%) and long τ_p_ (exp. 4.4 µs), respectively. These SOC constants ($\langle {{\mathrm{S}}_2}| {{\mathrm{\hat H}}soc} |{{\mathrm{T}}_{4,3,2}}\rangle $ and $\langle {{\mathrm{T}}_{1,2}}| {{\mathrm{\hat H}}soc} |{{\mathrm{S}}_0}\rangle $) in **Au_13_** are enhanced up to 80∼130 cm^−1^, which is responsible for the short τ_p_ and high Φ_p_. Turning to **Au_1_Ag_12_**, a significant charge transfer (CT) character is observed in the T_1_ states (*D* index* *= 4.73 Å), in addition to a predominant CT nature for their S_1_ states (*D* index* *= 5.19 Å). Consequently, Δ*E*_ST_ is small (∼0.01 eV), but $\langle {{\mathrm{S}}_1}| {{\mathrm{\hat H}}soc} |{{\mathrm{T}}_1}\rangle $ (23.97 cm^−1^) and $\langle {{\mathrm{T}}_1}| {{\mathrm{\hat H}}soc} |{{\mathrm{S}}_0}\rangle $ (40.49 cm^−1^) <41 cm^−1^, thus leading to an increase in Φ_p_ (exp. 9%) and decrease in τ_p_ (exp. 3.8 µs), respectively. Owing to the change in transition characters of the singlet and triplet states in the **Au_3_Ag_10_**cluster, $\langle {{\mathrm{S}}_1}| {{\mathrm{\hat H}}soc} |{{\mathrm{T}}_1}\rangle $ is enhanced up to 45.55 cm^−1^, and $\langle {{\mathrm{T}}_1}| {{\mathrm{\hat H}}soc} |{{\mathrm{S}}_0}\rangle $ up to 213.50 cm^−1^, respectively. On the other hand, the CT triplet state becomes energetically close to the LE triplet state (0.03 eV), which is responsible for the short τ_p_ (exp. 5.2 µs) and high Φ_p_ (exp. 38%). The emission and UV absorption spectra of M_13_ in different solvents further confirm that the emission of **Au_13_** originates from the LE state, while the emission of alloy clusters originates from the CT state (Figs S32 and S33) [76–78]. On the other hand, **Au_13_** exhibits a large structural change from T_1_/T_2_ to S_0_, as shown in Fig. S34, whereas **Ag_13_, Au_1_Ag_12_** and **Au_3_Ag_10_** retain nearly the same structure for the T_1_ and S_0_ states. For **Au_13_**, its structural deformation comes mainly from the core. A detailed comparison of the bond lengths of the equilibrium structure of the S_0_, S_1_/S_2_, and T_1_/T_2_ states of **Au*_n_*Ag_13−_*_n_*** (*n* = 0, 1, 3, 13) is listed in Tables S6–S9. The large structural relaxation of **Au_13_** leads to more energy dissipation.

**Figure 4.**
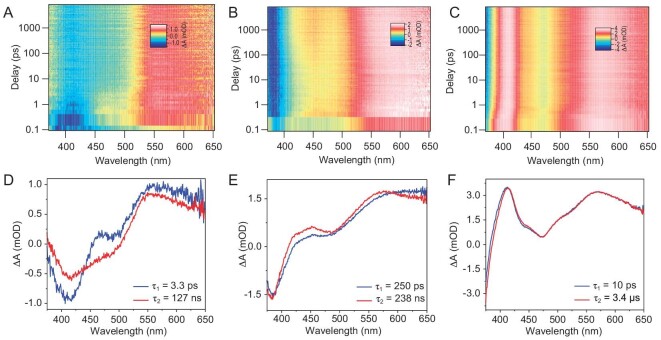
TA data maps of (A) **Ag_13_**, (B) **Au*_n_*Ag_13−_*_n_*** (10%), and (C) **Au_13_** (pumped at 360 nm in DMSO). TA spectra of (D) **Ag_13_**, (E) **Au*_n_*Ag_13−_*_n_*** (10%) and (F) **Au_13_** at two different time delays.

Femtosecond-nanosecond transient absorption (fs-ns TA) was used to deeply analyze the excited state behavior of **Ag_13_, Au*_n_*Ag_13−_*_n_*** and **Au_13_**. After irradiating by a 360 nm laser pulse, the excited electrons in the high energy state (403 nm) were reduced for **Ag_13_** (Fig. 4A) while increased for **Au*_n_*Ag_13−_*_n_*** (Fig. 4B, and Figs S35A, S36A) and **Au_13_** (Fig. 4C), indicating that there was an electron transfer process between them. The ground state bleaching (GSB) signal absorption of **Ag_13_** (Fig. 4D) and alloy clusters (Figs S35B, S36B) can be observed. As for **Au_13_**, although no corresponding GSB absorption was observed at 348 nm due to the selection of laser pulse, the change of its trajectory also indicated that it has a set of negative signals corresponding to the absorption (Fig. 4F). The non-negative signals at ∼480 nm may be caused by the overlap between excited state absorption (ESA) and GSB at 500 nm. The global fitting analysis demonstrated that **Ag_13_** had a decay constant of 3.3 ps and 127 ns (Fig. 4D), in which the 3.3 ps process was assigned to IC. The 127 ns process was attributed to the return of the excited electrons from the T_1_ state to the S_0_ state. However, **Ag_13_** has a fast non-radiative transition in solution, so the luminescence is quenched and the triplet state lifetime is short. Moreover, the decay constants of the 10% alloy cluster and **Au_13_** were 250 ps, 238 ns (Fig. 4E), and 10 ps, 3.4 µs (Fig. 4F), respectively. Combined with DFT and TD-DFT calculation results, we believe that the 250 ps and 238 ns process of 10% alloy cluster can be attributable to the electron transfer from the ligand to metal core (LMCT) and electron return to the ground state after reaching a newly excited state through LMCT, respectively. The 10 ps process of **Au_13_** is considered to be a slower IC process. The process of 3.4 μs belongs to the electron reaching the newly excited state and finally returning to the ground state via radiative transition, producing luminescence with a high quantum yield.

**Figure 5.**
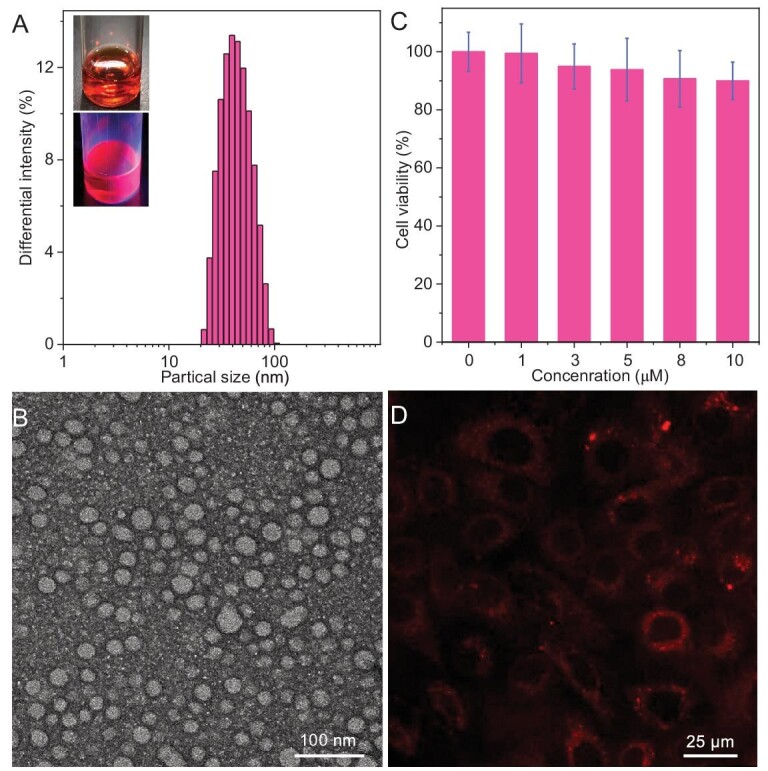
(A) Dynamic light scattering results of **Au_13_@PLGA** in PBS. Inset: images of **Au_13_@PLGA** under natural light (top) and UV (bottom) irradiation. (B) TEM image of **Au_13_@PLGA**. (C) Viability of HeLa cells after treatment with different concentrations of **Au_13_@PLGA** for 24 h. (D) Confocal image of HeLa cells incubated with **Au_13_@PLGA** for 2 h.

Due to its NIR photoluminescence with an ultrahigh QY feature, the **Au_13_** clusters produced can be utilized for bioimaging. First, we employed mPEG-PLGA to encapsulate **Au_13_** clusters to enhance their water dispersibility and biocompatibility. As shown in Fig. 5A, the **Au_13_** nanoclusters aggregated into uniform nano-micelles (**Au_13_@PLGA**) with an average particle size of ∼49.2 nm in Dulbecco’s phosphate-buffered saline (PBS). In addition, the emission spectrum of **Au_13_@PLGA** still exhibits excellent NIR photoluminescence, similar to that of **Au_13_**, indicating that the encapsulation of PLGA does not deteriorate the structure of **Au_13_** (Fig. S37)_._ Moreover, the transmission electron microscopy (TEM) images further demonstrated that free **Au_13_** (∼1 nm, Fig. S38) was assembled with mPEG-PLGA to form uniform nanoparticles with sizes of 30–50 nm (Fig. 5B). After incubating with different concentrations (from 0 to 10 μM) of **Au_13_@PLGA** for 24 h, the survival rate of HeLa cells exceeded 90% (Fig. 5C), indicating its good biocompatibility. **Au_13_@PLGA** (4 μM) was then used for cell imaging. The confocal image exhibited significant intracellular red luminescence in HeLa cells after culturing for 2 h (Fig. 5D), indicating **Au_13_** was a promising NIR biological imaging reagent.

## CONCLUSION

In summary, we synthesized a pair of gold-silver analogue clusters (**Ag_13_** and **Au_13_**) as well as their alloy clusters (**Au*_n_*Ag_13−_*_n_***), where the icosahedral **Ag_13_** was first reported as a whole rather than as a kernel. The **Ag_13_** solution exhibited non-luminescence, while the analogue **Au_13_** solution displayed bright NIR luminescence with a QY up to 45%. The DFT calculation shows that the evolution of **Ag_13_** and **Au_13_** leads to the change of nanoclusters’ energy level and structural rigidity with the increase of gold atom content, which leads to the subsequent variation of optical properties of **M_13_** series nanoclusters. This work reported the icosahedral **Ag_13_** structure for the first time and the evolution from **Ag_13_** to the analogous nanocluster **Au_13_**, uncovering the changes in optical properties during this process and revealing the intrinsic underlying causes.

## Supplementary Material

nwae174_Supplemental_Files
