# Supplementary material for: Filling the gaps in icosahedral superatomic metal clusters
Source: Natl Sci Rev. 2024 May 28;11(7):nwae174. doi: 10.1093/nsr/nwae174 (PMC11182670; doi:10.1093/nsr/nwae174)

checkCIF/PLATON report

Structure factors have been supplied for datablock(s) ag13

THIS REPORT IS FOR GUIDANCE ONLY. IF USED AS PART OF A REVIEW PROCEDURE FOR PUBLICATION, IT SHOULD NOT REPLACE THE EXPERTISE OF AN EXPERIENCED CRYSTALLOGRAPHIC REFEREE.

No syntax errors found. CIF dictionary Interpreting this report

Datablock: ag13

Bond precision: C-C = 0.0352 A Wavelength=1.54184

Cell:	a=19.2451(3)	b=20.2312(3)	c=22.8333(4)
	alpha=97.183(1)	beta=99.545(1)	gamma=109.667(1)
Temperature:	200 K		

	Calculated	Reported
Volume	8097.6(2)	8097.6(2)
Space group	P -1	P -1
Hall group	-P 1	-P 1
Moiety formula	C145 H115 Ag13 Cl2 N5 P10, 3(F6 Sb)	3(F6 Sb), C145 H115 Ag13 Cl2 N5 P10
Sum formula	C145 H115 Ag13 Cl2 F18 N5 P10 Sb3	C145 H115 Ag13 Cl2 F18 N5 P10 Sb3
Mr	4417.62	4417.57
Dx,g cm-3	1.812	1.812
Z	2	2
Mu (mm-1)	17.989	17.989
F000	4260.0	4260.0
F000'	4281.33	
h,k,lmax	23,25,28	23,25,28
Nref	32792	31694
Tmin,Tmax	0.391,0.487	0.215,1.000
Tmin'	0.295	

Correction method= # Reported T Limits: Tmin=0.215 Tmax=1.000
AbsCorr = MULTI-SCAN

Data completeness= 0.967 Theta(max)= 73.847

```
R(reflections)= 0.0947( 25888)          wR2(reflections)= 0.2447( 31694)
S = 1.089                    Npar= 1902
```

The following ALERTS were generated. Each ALERT has the format
test-name_ALERT_alert-type_alert-level.
Click on the hyperlinks for more details of the test.

Alert level A

```
PLAT602_ALERT_2_A Solvent Accessible VOID(S) in Structure ........          ! Check
```

Alert level B

```
PLAT342_ALERT_3_B Low Bond Precision on  C-C Bonds ..............    0.03523 Ang.
```

Alert level C

```
PLAT042_ALERT_1_C Calc. and Reported MoietyFormula Strings  Differ     Please Check
PLAT220_ALERT_2_C NonSolvent   Resd 1  C   Ueq(max)/Ueq(min) Range        4.6 Ratio
PLAT234_ALERT_4_C Large Hirshfeld Difference P7       --C94      .       0.18 Ang.
PLAT234_ALERT_4_C Large Hirshfeld Difference N5       --C138     .       0.17 Ang.
PLAT234_ALERT_4_C Large Hirshfeld Difference C24      --C25      .       0.16 Ang.
PLAT234_ALERT_4_C Large Hirshfeld Difference C50      --C51      .       0.22 Ang.
PLAT234_ALERT_4_C Large Hirshfeld Difference C60      --C61      .       0.21 Ang.
PLAT234_ALERT_4_C Large Hirshfeld Difference C63      --C64      .       0.19 Ang.
PLAT234_ALERT_4_C Large Hirshfeld Difference C77      --C78      .       0.19 Ang.
PLAT234_ALERT_4_C Large Hirshfeld Difference C80      --C81      .       0.23 Ang.
PLAT234_ALERT_4_C Large Hirshfeld Difference C111     --C113     .       0.24 Ang.
PLAT234_ALERT_4_C Large Hirshfeld Difference Sb1      --F3       .       0.19 Ang.
PLAT234_ALERT_4_C Large Hirshfeld Difference Sb1      --F6       .       0.23 Ang.
PLAT234_ALERT_4_C Large Hirshfeld Difference Sb2      --F1       .       0.18 Ang.
PLAT234_ALERT_4_C Large Hirshfeld Difference Sb2      --F2       .       0.18 Ang.
PLAT234_ALERT_4_C Large Hirshfeld Difference Sb2      --F3       .       0.17 Ang.
PLAT234_ALERT_4_C Large Hirshfeld Difference Sb2      --F6       .       0.25 Ang.
PLAT234_ALERT_4_C Large Hirshfeld Difference Sb3      --F7       .       0.21 Ang.
PLAT234_ALERT_4_C Large Hirshfeld Difference Sb3      --F9       .       0.19 Ang.
PLAT234_ALERT_4_C Large Hirshfeld Difference Sb3      --F10      .       0.18 Ang.
PLAT241_ALERT_2_C High   'MainMol' Ueq as Compared to Neighbors of        C5 Check
PLAT241_ALERT_2_C High   'MainMol' Ueq as Compared to Neighbors of       C28 Check
PLAT241_ALERT_2_C High   'MainMol' Ueq as Compared to Neighbors of       C49 Check
PLAT241_ALERT_2_C High   'MainMol' Ueq as Compared to Neighbors of       C51 Check
PLAT241_ALERT_2_C High   'MainMol' Ueq as Compared to Neighbors of       C61 Check
PLAT241_ALERT_2_C High   'MainMol' Ueq as Compared to Neighbors of       C63 Check
PLAT241_ALERT_2_C High   'MainMol' Ueq as Compared to Neighbors of       C67 Check
PLAT241_ALERT_2_C High   'MainMol' Ueq as Compared to Neighbors of       C80 Check
PLAT241_ALERT_2_C High   'MainMol' Ueq as Compared to Neighbors of      C106 Check
PLAT241_ALERT_2_C High   'MainMol' Ueq as Compared to Neighbors of      C113 Check
PLAT241_ALERT_2_C High   'MainMol' Ueq as Compared to Neighbors of      C140 Check
PLAT242_ALERT_2_C Low    'MainMol' Ueq as Compared to Neighbors of       C76 Check
PLAT243_ALERT_4_C High   'Solvent' Ueq as Compared to Neighbors of        F1 Check
PLAT243_ALERT_4_C High   'Solvent' Ueq as Compared to Neighbors of        F2 Check
PLAT243_ALERT_4_C High   'Solvent' Ueq as Compared to Neighbors of        F3 Check
PLAT243_ALERT_4_C High   'Solvent' Ueq as Compared to Neighbors of        F4 Check
PLAT243_ALERT_4_C High   'Solvent' Ueq as Compared to Neighbors of        F5 Check
```

```
PLAT243_ALERT_4_C High   'Solvent' Ueq as Compared to Neighbors of         F6 Check
PLAT244_ALERT_4_C Low    'Solvent' Ueq as Compared to Neighbors of        Sb3 Check
PLAT260_ALERT_2_C Large Average Ueq of Residue Including       Sb3      0.105 Check
PLAT260_ALERT_2_C Large Average Ueq of Residue Including       Sb4      0.124 Check
PLAT260_ALERT_2_C Large Average Ueq of Residue Including       Sb5      0.132 Check
PLAT332_ALERT_2_C Large Phenyl C-C Range     C126      -C131     .       0.17 Ang.
PLAT906_ALERT_3_C Large K Value in the Analysis of Variance ......     14.405 Check
PLAT906_ALERT_3_C Large K Value in the Analysis of Variance ......      3.664 Check
PLAT911_ALERT_3_C Missing FCF Refl Between Thmin & STh/L=    0.600        199 Report
PLAT918_ALERT_3_C Reflection(s) with I(obs) much Smaller I(calc) .          1 Check
```

Alert level G

```
PLAT002_ALERT_2_G Number of Distance or Angle Restraints on AtSite         29 Note
PLAT003_ALERT_2_G Number of Uiso or Uij Restrained non-H Atoms ...         73 Report
PLAT083_ALERT_2_G SHELXL Second Parameter in WGHT  Unusually Large     350.20 Why ?
PLAT154_ALERT_1_G The s.u.'s on the Cell Angles are Equal ..(Note)      0.001 Degree
PLAT172_ALERT_4_G The CIF-Embedded .res File Contains DFIX Records          2 Report
PLAT175_ALERT_4_G The CIF-Embedded .res File Contains SAME Records          1 Report
PLAT176_ALERT_4_G The CIF-Embedded .res File Contains SADI Records          5 Report
PLAT177_ALERT_4_G The CIF-Embedded .res File Contains DELU Records          6 Report
PLAT186_ALERT_4_G The CIF-Embedded .res File Contains ISOR Records         11 Report
PLAT233_ALERT_4_G Hirshfeld (M-X Solvent)    Sb1      --F4       .        9.8 s.u.
PLAT233_ALERT_4_G Hirshfeld (M-X Solvent)    Sb1      --F5       .        8.8 s.u.
PLAT233_ALERT_4_G Hirshfeld (M-X Solvent)    Sb2      --F4       .        9.3 s.u.
PLAT233_ALERT_4_G Hirshfeld (M-X Solvent)    Sb2      --F5       .        8.4 s.u.
PLAT301_ALERT_3_G Main Residue  Disorder .............(Resd  1  )         5% Note
PLAT302_ALERT_4_G Anion/Solvent/Minor-Residue Disorder (Resd  2  )        14% Note
PLAT302_ALERT_4_G Anion/Solvent/Minor-Residue Disorder (Resd  4  )       100% Note
PLAT302_ALERT_4_G Anion/Solvent/Minor-Residue Disorder (Resd  5  )       100% Note
PLAT304_ALERT_4_G Non-Integer Number of Atoms in ..... (Resd  4  )       3.50 Check
PLAT304_ALERT_4_G Non-Integer Number of Atoms in ..... (Resd  5  )       3.50 Check
PLAT410_ALERT_2_G Short Intra H...H Contact  H108      ..H122     .       2.14 Ang.
                                                      x,y,z  =      1_555 Check
PLAT721_ALERT_1_G Bond    Calc     0.93000, Rep     0.95000 Dev...       0.02 Ang.
              C124     -H124           1_555   1_555 ........     #  345 Check
PLAT722_ALERT_1_G Angle   Calc      119.00, Rep      117.90 Dev...       1.10 Degree
              C94     -C95     -H95        1_555  1_555  1_555     #  739 Check
PLAT722_ALERT_1_G Angle   Calc      123.00, Rep      121.80 Dev...       1.20 Degree
              C99     -C98     -H98        1_555  1_555  1_555     #  749 Check
PLAT722_ALERT_1_G Angle   Calc      120.00, Rep      121.20 Dev...       1.20 Degree
              C121    -C120    -H120       1_555  1_555  1_555     #  823 Check
PLAT722_ALERT_1_G Angle   Calc      124.00, Rep      122.90 Dev...       1.10 Degree
              C125    -C124    -H124       1_555  1_555  1_555     #  835 Check
PLAT790_ALERT_4_G Centre of Gravity not Within Unit Cell: Resd.  #          3 Note
              F6 Sb
PLAT790_ALERT_4_G Centre of Gravity not Within Unit Cell: Resd.  #          4 Note
              F6 Sb
PLAT790_ALERT_4_G Centre of Gravity not Within Unit Cell: Resd.  #          5 Note
              F6 Sb
PLAT793_ALERT_4_G Model has Chirality at P1          (Centro SPGR)          S Verify
PLAT793_ALERT_4_G Model has Chirality at P4          (Centro SPGR)          S Verify
PLAT793_ALERT_4_G Model has Chirality at P8          (Centro SPGR)          S Verify
PLAT793_ALERT_4_G Model has Chirality at P9          (Centro SPGR)          S Verify
PLAT793_ALERT_4_G Model has Chirality at P10         (Centro SPGR)          R Verify
PLAT794_ALERT_5_G Tentative Bond Valency for Sb3       (V)       .       5.38 Info
PLAT860_ALERT_3_G Number of Least-Squares Restraints ............         623 Note
```

```
PLAT870_ALERT_4_G ALERTS Related to Twinning Effects Suppressed ..          ! Info
PLAT883_ALERT_1_G No Info/Value for _atom_sites_solution_primary .     Please Do !
PLAT910_ALERT_3_G Missing # of FCF Reflection(s) Below Theta(Min).          3 Note
PLAT912_ALERT_4_G Missing # of FCF Reflections Above STh/L=  0.600        896 Note
PLAT913_ALERT_3_G Missing # of Very Strong Reflections in FCF ....          2 Note
PLAT931_ALERT_5_G CIFcalcFCF  Twin Law ( 1-1 0)         Est.d BASF       0.20 Check
PLAT933_ALERT_2_G Number of HKL-OMIT Records in Embedded .res File         12 Note
PLAT941_ALERT_3_G Average HKL Measurement Multiplicity ..........         1.0 Low
```

```
   1 ALERT level A = Most likely a serious problem - resolve or explain
   1 ALERT level B = A potentially serious problem, consider carefully
  47 ALERT level C = Check. Ensure it is not caused by an omission or oversight
  43 ALERT level G = General information/check it is not something unexpected

   8 ALERT type 1 CIF construction/syntax error, inconsistent or missing data
  23 ALERT type 2 Indicator that the structure model may be wrong or deficient
  10 ALERT type 3 Indicator that the structure quality may be low
  49 ALERT type 4 Improvement, methodology, query or suggestion
   2 ALERT type 5 Informative message, check
```

It is advisable to attempt to resolve as many as possible of the alerts in all categories. Often the minor alerts point to easily fixed oversights, errors and omissions in your CIF or refinement strategy, so attention to these fine details can be worthwhile. In order to resolve some of the more serious problems it may be necessary to carry out additional measurements or structure refinements. However, the purpose of your study may justify the reported deviations and the more serious of these should normally be commented upon in the discussion or experimental section of a paper or in the "special_details" fields of the CIF. checkCIF was carefully designed to identify outliers and unusual parameters, but every test has its limitations and alerts that are not important in a particular case may appear. Conversely, the absence of alerts does not guarantee there are no aspects of the results needing attention. It is up to the individual to critically assess their own results and, if necessary, seek expert advice.

Publication of your CIF in IUCr journals

A basic structural check has been run on your CIF. These basic checks will be run on all CIFs submitted for publication in IUCr journals (*Acta Crystallographica*, *Journal of Applied Crystallography*, *Journal of Synchrotron Radiation*); however, if you intend to submit to *Acta Crystallographica Section C* or *E* or *IUCrData*, you should make sure that full publication checks are run on the final version of your CIF prior to submission.

Publication of your CIF in other journals

Please refer to the *Notes for Authors* of the relevant journal for any special instructions relating to CIF submission.

PLATON version of 18/05/2022; check.def file version of 17/05/2022

Datablock ag13 - ellipsoid plot

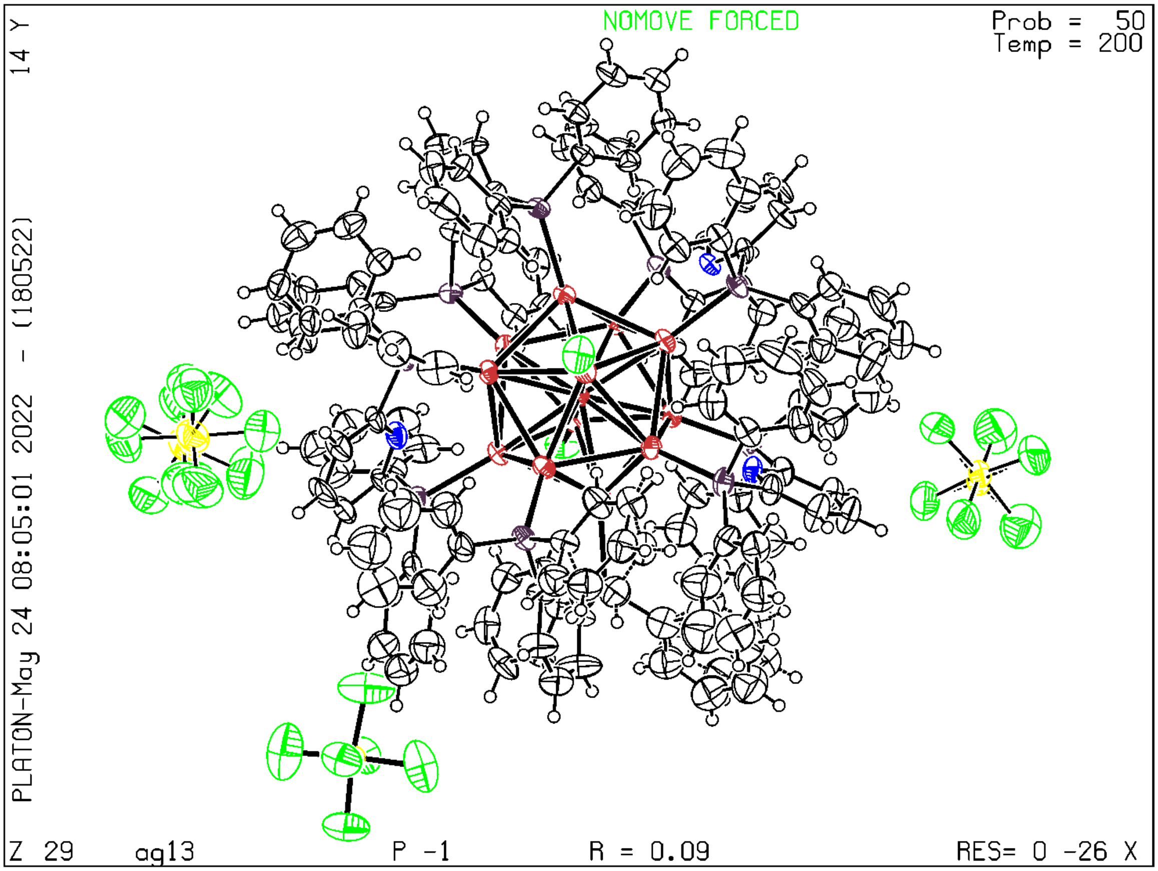

Supplement: nwae174_Supplemental_Files [file nwae174_supplemental_files.zip › Ag13-checkcif.pdf]
