# Supplementary material for: Filling the gaps in icosahedral superatomic metal clusters
Source: Natl Sci Rev. 2024 May 28;11(7):nwae174. doi: 10.1093/nsr/nwae174 (PMC11182670; doi:10.1093/nsr/nwae174)

checkCIF/PLATON report

You have not supplied any structure factors. As a result the full set of tests cannot be run.

THIS REPORT IS FOR GUIDANCE ONLY. IF USED AS PART OF A REVIEW PROCEDURE FOR PUBLICATION, IT SHOULD NOT REPLACE THE EXPERTISE OF AN EXPERIENCED CRYSTALLOGRAPHIC REFEREE.

No syntax errors found. CIF dictionary Interpreting this report

Datablock: au13

```
Bond precision:   C-C = 0.0287 A           Wavelength=1.54184

Cell:           a=22.3598(1)       b=24.1935(2)      c=34.4724(3)
                alpha=71.818(1)    beta=79.021(1)    gamma=88.162(1)
Temperature:    200 K
```

	Calculated	Reported
Volume	17385.4(3)	17385.4(2)
Space group	P -1	P -1
Hall group	-P 1	-P 1
Moiety formula	C145 H115 Au13 Cl2 N5 P10, 3(F6 Sb) [+ solvent]	C145 H115 Au13 Cl2 N5 P10, 3(F6 Sb)
Sum formula	C145 H115 Au13 Cl2 F18 N5 P10 Sb3 [+ solvent]	C145 H115 Au13 Cl2 F18 N5 P10 Sb3
Mr	5575.88	5575.83
Dx,g cm-3	2.130	2.130
Z	4	4
Mu (mm-1)	25.341	25.341
F000	10184.0	10184.0
F000’	9993.34	
h,k,lmax	27,30,42	26,30,42
Nref	70445	68048
Tmin,Tmax	0.119,0.079	0.003,1.000
Tmin’	0.019	

```
Correction method= # Reported T Limits: Tmin=0.003 Tmax=1.000
AbsCorr = MULTI-SCAN

Data completeness= 0.966            Theta(max)= 73.855
```

R(reflections)= 0.0808(52652)   wR2(reflections)= 0.2395(68048)

S = 1.052   Npar= 3752

The following ALERTS were generated. Each ALERT has the format
test-name_ALERT_alert-type_alert-level.
Click on the hyperlinks for more details of the test.

Alert level B

PLAT342_ALERT_3_B Low Bond Precision on C-C Bonds .... 0.02869 Ang.

Alert level C

DIFMX02_ALERT_1_C The maximum difference density is > 0.1*ZMAX*0.75
The relevant atom site should be identified.
PLAT097_ALERT_2_C Large Reported Max. (Positive) Residual Density 6.28 eA-3
PLAT213_ALERT_2_C Atom C14 has ADP max/min Ratio ..... 3.9 oblate
PLAT213_ALERT_2_C Atom C107 has ADP max/min Ratio ..... 3.2 oblate
PLAT220_ALERT_2_C NonSolvent Resd 1 C Ueq(max)/Ueq(min) Range 3.1 Ratio
PLAT220_ALERT_2_C NonSolvent Resd 2 C Ueq(max)/Ueq(min) Range 3.2 Ratio
PLAT241_ALERT_2_C High 'MainMol' Ueq as Compared to Neighbors of C11 Check
PLAT241_ALERT_2_C High 'MainMol' Ueq as Compared to Neighbors of C15 Check
PLAT241_ALERT_2_C High 'MainMol' Ueq as Compared to Neighbors of C25 Check
PLAT241_ALERT_2_C High 'MainMol' Ueq as Compared to Neighbors of C29 Check
PLAT241_ALERT_2_C High 'MainMol' Ueq as Compared to Neighbors of C57 Check
PLAT241_ALERT_2_C High 'MainMol' Ueq as Compared to Neighbors of C68 Check
PLAT241_ALERT_2_C High 'MainMol' Ueq as Compared to Neighbors of C79 Check
PLAT241_ALERT_2_C High 'MainMol' Ueq as Compared to Neighbors of C86 Check
PLAT241_ALERT_2_C High 'MainMol' Ueq as Compared to Neighbors of C89 Check
PLAT241_ALERT_2_C High 'MainMol' Ueq as Compared to Neighbors of C92 Check
PLAT241_ALERT_2_C High 'MainMol' Ueq as Compared to Neighbors of C98 Check
PLAT241_ALERT_2_C High 'MainMol' Ueq as Compared to Neighbors of C102 Check
PLAT241_ALERT_2_C High 'MainMol' Ueq as Compared to Neighbors of C107 Check
PLAT241_ALERT_2_C High 'MainMol' Ueq as Compared to Neighbors of C114 Check
PLAT241_ALERT_2_C High 'MainMol' Ueq as Compared to Neighbors of C141 Check
PLAT241_ALERT_2_C High 'MainMol' Ueq as Compared to Neighbors of C148 Check
PLAT241_ALERT_2_C High 'MainMol' Ueq as Compared to Neighbors of C159 Check
PLAT241_ALERT_2_C High 'MainMol' Ueq as Compared to Neighbors of C165 Check
PLAT241_ALERT_2_C High 'MainMol' Ueq as Compared to Neighbors of C172 Check
PLAT241_ALERT_2_C High 'MainMol' Ueq as Compared to Neighbors of C177 Check
PLAT241_ALERT_2_C High 'MainMol' Ueq as Compared to Neighbors of C189 Check
PLAT241_ALERT_2_C High 'MainMol' Ueq as Compared to Neighbors of C205 Check
PLAT241_ALERT_2_C High 'MainMol' Ueq as Compared to Neighbors of C207 Check
PLAT241_ALERT_2_C High 'MainMol' Ueq as Compared to Neighbors of C218 Check
PLAT241_ALERT_2_C High 'MainMol' Ueq as Compared to Neighbors of C229 Check
PLAT241_ALERT_2_C High 'MainMol' Ueq as Compared to Neighbors of C264 Check
PLAT241_ALERT_2_C High 'MainMol' Ueq as Compared to Neighbors of C266 Check
PLAT241_ALERT_2_C High 'MainMol' Ueq as Compared to Neighbors of C283 Check
PLAT241_ALERT_2_C High 'MainMol' Ueq as Compared to Neighbors of C288 Check
PLAT242_ALERT_2_C Low 'MainMol' Ueq as Compared to Neighbors of C10 Check
PLAT242_ALERT_2_C Low 'MainMol' Ueq as Compared to Neighbors of C24 Check
PLAT242_ALERT_2_C Low 'MainMol' Ueq as Compared to Neighbors of C93 Check
PLAT242_ALERT_2_C Low 'MainMol' Ueq as Compared to Neighbors of C105 Check
PLAT242_ALERT_2_C Low 'MainMol' Ueq as Compared to Neighbors of C109 Check

```
PLAT242_ALERT_2_C Low    'MainMol' Ueq as Compared to Neighbors of       C116 Check
PLAT242_ALERT_2_C Low    'MainMol' Ueq as Compared to Neighbors of       C123 Check
PLAT242_ALERT_2_C Low    'MainMol' Ueq as Compared to Neighbors of       C163 Check
PLAT242_ALERT_2_C Low    'MainMol' Ueq as Compared to Neighbors of       C175 Check
PLAT242_ALERT_2_C Low    'MainMol' Ueq as Compared to Neighbors of       C204 Check
PLAT242_ALERT_2_C Low    'MainMol' Ueq as Compared to Neighbors of       C206 Check
PLAT242_ALERT_2_C Low    'MainMol' Ueq as Compared to Neighbors of       C262 Check
PLAT242_ALERT_2_C Low    'MainMol' Ueq as Compared to Neighbors of       C263 Check
PLAT242_ALERT_2_C Low    'MainMol' Ueq as Compared to Neighbors of       C265 Check
PLAT244_ALERT_4_C Low    'Solvent' Ueq as Compared to Neighbors of        Sb3 Check
PLAT244_ALERT_4_C Low    'Solvent' Ueq as Compared to Neighbors of        Sb4 Check
PLAT250_ALERT_2_C Large U3/U1 Ratio for Average U(i,j) Tensor ....        2.5 Note
PLAT250_ALERT_2_C Large U3/U1 Ratio for Average U(i,j) Tensor ....        2.8 Note
PLAT250_ALERT_2_C Large U3/U1 Ratio for Average U(i,j) Tensor ....        2.4 Note
PLAT260_ALERT_2_C Large Average Ueq of Residue Including       Sb1      0.120 Check
PLAT260_ALERT_2_C Large Average Ueq of Residue Including       Sb2      0.180 Check
PLAT260_ALERT_2_C Large Average Ueq of Residue Including       Sb3      0.132 Check
PLAT260_ALERT_2_C Large Average Ueq of Residue Including       Sb4      0.183 Check
PLAT260_ALERT_2_C Large Average Ueq of Residue Including       Sb5      0.143 Check
PLAT260_ALERT_2_C Large Average Ueq of Residue Including       Sb6      0.130 Check
PLAT260_ALERT_2_C Large Average Ueq of Residue Including      Sb5A      0.104 Check
PLAT260_ALERT_2_C Large Average Ueq of Residue Including       Sb7      0.133 Check
PLAT332_ALERT_2_C Large Phenyl C-C Range     C7        -C12       .        0.24 Ang.
PLAT332_ALERT_2_C Large Phenyl C-C Range     C24       -C29       .        0.20 Ang.
PLAT332_ALERT_2_C Large Phenyl C-C Range     C53       -C58       .        0.19 Ang.
PLAT332_ALERT_2_C Large Phenyl C-C Range     C105      -C110      .        0.20 Ang.
PLAT332_ALERT_2_C Large Phenyl C-C Range     C111      -C116      .        0.16 Ang.
PLAT332_ALERT_2_C Large Phenyl C-C Range     C204      -C209      .        0.22 Ang.
PLAT332_ALERT_2_C Large Phenyl C-C Range     C210      -C215      .        0.17 Ang.
PLAT332_ALERT_2_C Large Phenyl C-C Range     C239      -C244      .        0.18 Ang.
```

Alert level G

```
PLAT002_ALERT_2_G Number of Distance or Angle Restraints on AtSite         89 Note
PLAT003_ALERT_2_G Number of Uiso or Uij Restrained non-H Atoms ...        158 Report
PLAT072_ALERT_2_G SHELXL First  Parameter in WGHT  Unusually Large       0.17 Report
PLAT154_ALERT_1_G The s.u.'s on the Cell Angles are Equal ..(Note)      0.001 Degree
PLAT172_ALERT_4_G The CIF-Embedded .res File Contains DFIX Records          5 Report
PLAT175_ALERT_4_G The CIF-Embedded .res File Contains SAME Records          9 Report
PLAT176_ALERT_4_G The CIF-Embedded .res File Contains SADI Records          6 Report
PLAT177_ALERT_4_G The CIF-Embedded .res File Contains DELU Records         12 Report
PLAT178_ALERT_4_G The CIF-Embedded .res File Contains SIMU Records          4 Report
PLAT186_ALERT_4_G The CIF-Embedded .res File Contains ISOR Records         23 Report
PLAT300_ALERT_4_G Atom Site Occupancy of Sb1        Constrained at       0.85 Check
PLAT300_ALERT_4_G Atom Site Occupancy of F1         Constrained at       0.85 Check
PLAT300_ALERT_4_G Atom Site Occupancy of F2         Constrained at       0.85 Check
PLAT300_ALERT_4_G Atom Site Occupancy of F3         Constrained at       0.85 Check
PLAT300_ALERT_4_G Atom Site Occupancy of F4         Constrained at       0.85 Check
PLAT300_ALERT_4_G Atom Site Occupancy of F5         Constrained at       0.85 Check
PLAT300_ALERT_4_G Atom Site Occupancy of F6         Constrained at       0.85 Check
PLAT300_ALERT_4_G Atom Site Occupancy of Sb2        Constrained at        0.5 Check
PLAT300_ALERT_4_G Atom Site Occupancy of F7         Constrained at        0.5 Check
PLAT300_ALERT_4_G Atom Site Occupancy of F8         Constrained at        0.5 Check
PLAT300_ALERT_4_G Atom Site Occupancy of F9         Constrained at        0.5 Check
PLAT300_ALERT_4_G Atom Site Occupancy of F10        Constrained at        0.5 Check
PLAT300_ALERT_4_G Atom Site Occupancy of F11        Constrained at        0.5 Check
PLAT300_ALERT_4_G Atom Site Occupancy of F12        Constrained at        0.5 Check
```

```
PLAT300_ALERT_4_G Atom Site Occupancy of Sb1A       Constrained at       0.15 Check
PLAT300_ALERT_4_G Atom Site Occupancy of F1A        Constrained at       0.15 Check
PLAT300_ALERT_4_G Atom Site Occupancy of F2A        Constrained at       0.15 Check
PLAT300_ALERT_4_G Atom Site Occupancy of F3A        Constrained at       0.15 Check
PLAT300_ALERT_4_G Atom Site Occupancy of F4A        Constrained at       0.15 Check
PLAT300_ALERT_4_G Atom Site Occupancy of F5A        Constrained at       0.15 Check
PLAT300_ALERT_4_G Atom Site Occupancy of F6A        Constrained at       0.15 Check
PLAT302_ALERT_4_G Anion/Solvent/Minor-Residue Disorder (Resd  3  )       100% Note
PLAT302_ALERT_4_G Anion/Solvent/Minor-Residue Disorder (Resd  4  )       100% Note
PLAT302_ALERT_4_G Anion/Solvent/Minor-Residue Disorder (Resd  7  )       100% Note
PLAT302_ALERT_4_G Anion/Solvent/Minor-Residue Disorder (Resd  8  )       100% Note
PLAT302_ALERT_4_G Anion/Solvent/Minor-Residue Disorder (Resd  9  )       100% Note
PLAT302_ALERT_4_G Anion/Solvent/Minor-Residue Disorder (Resd 10  )       100% Note
PLAT302_ALERT_4_G Anion/Solvent/Minor-Residue Disorder (Resd 11  )       100% Note
PLAT302_ALERT_4_G Anion/Solvent/Minor-Residue Disorder (Resd 12  )       100% Note
PLAT302_ALERT_4_G Anion/Solvent/Minor-Residue Disorder (Resd 13  )       100% Note
PLAT304_ALERT_4_G Non-Integer Number of Atoms in ..... (Resd  3  )       5.95 Check
PLAT304_ALERT_4_G Non-Integer Number of Atoms in ..... (Resd  4  )       3.50 Check
PLAT304_ALERT_4_G Non-Integer Number of Atoms in ..... (Resd  7  )       5.46 Check
PLAT304_ALERT_4_G Non-Integer Number of Atoms in ..... (Resd  8  )       3.77 Check
PLAT304_ALERT_4_G Non-Integer Number of Atoms in ..... (Resd  9  )       1.05 Check
PLAT304_ALERT_4_G Non-Integer Number of Atoms in ..... (Resd 10  )       1.54 Check
PLAT304_ALERT_4_G Non-Integer Number of Atoms in ..... (Resd 11  )       3.23 Check
PLAT304_ALERT_4_G Non-Integer Number of Atoms in ..... (Resd 12  )       2.06 Check
PLAT304_ALERT_4_G Non-Integer Number of Atoms in ..... (Resd 13  )       1.44 Check
PLAT432_ALERT_2_G Short Inter X...Y Contact  F37      ..C73              2.89 Ang.
                                                  x,y,z  =      1_555 Check
PLAT432_ALERT_2_G Short Inter X...Y Contact  F40      ..C229             2.93 Ang.
                                               x,-1+y,z  =      1_545 Check
PLAT432_ALERT_2_G Short Inter X...Y Contact  F40      ..C228             2.97 Ang.
                                               x,-1+y,z  =      1_545 Check
PLAT432_ALERT_2_G Short Inter X...Y Contact  F3A      ..C15              2.95 Ang.
                                             1-x,-y,1-z  =      2_656 Check
PLAT432_ALERT_2_G Short Inter X...Y Contact  F26A     ..C102             2.81 Ang.
                                                  x,y,z  =      1_555 Check
PLAT432_ALERT_2_G Short Inter X...Y Contact  F43      ..C142             2.92 Ang.
                                             -x,1-y,1-z  =      2_566 Check
PLAT432_ALERT_2_G Short Inter X...Y Contact  F43      ..C141             2.97 Ang.
                                             -x,1-y,1-z  =      2_566 Check
PLAT432_ALERT_2_G Short Inter X...Y Contact  F49      ..C56              2.84 Ang.
                                             -x,1-y,1-z  =      2_566 Check
PLAT432_ALERT_2_G Short Inter X...Y Contact  F50      ..C98              2.74 Ang.
                                                  x,y,z  =      1_555 Check
PLAT432_ALERT_2_G Short Inter X...Y Contact  F52      ..C57              2.42 Ang.
                                             -x,1-y,1-z  =      2_566 Check
PLAT434_ALERT_2_G Short Inter HL..HL Contact Cl4      ..Cl4              3.40 Ang.
                                            1-x,1-y,2-z  =      2_667 Check
PLAT434_ALERT_2_G Short Inter HL..HL Contact F18      ..F4A              2.79 Ang.
                                             1-x,-y,1-z  =      2_656 Check
PLAT606_ALERT_4_G Solvent Accessible VOID(S) in Structure ........          ! Info
PLAT790_ALERT_4_G Centre of Gravity not Within Unit Cell: Resd.  #          4 Note
              F6 Sb
PLAT790_ALERT_4_G Centre of Gravity not Within Unit Cell: Resd.  #          5 Note
              F6 Sb
PLAT790_ALERT_4_G Centre of Gravity not Within Unit Cell: Resd.  #          6 Note
              F6 Sb
PLAT790_ALERT_4_G Centre of Gravity not Within Unit Cell: Resd.  #          7 Note
```

```
              F6 Sb
PLAT790_ALERT_4_G Centre of Gravity not Within Unit Cell: Resd.  #          8 Note
              F6 Sb
PLAT790_ALERT_4_G Centre of Gravity not Within Unit Cell: Resd.  #         10 Note
              F6 Sb
PLAT790_ALERT_4_G Centre of Gravity not Within Unit Cell: Resd.  #         11 Note
              F6 Sb
PLAT790_ALERT_4_G Centre of Gravity not Within Unit Cell: Resd.  #         12 Note
              F6 Sb
PLAT790_ALERT_4_G Centre of Gravity not Within Unit Cell: Resd.  #         13 Note
              F6 Sb
PLAT794_ALERT_5_G Tentative Bond Valency for Sb3       (V)       .       5.26 Info
PLAT794_ALERT_5_G Tentative Bond Valency for Sb4       (V)       .       5.50 Info
PLAT860_ALERT_3_G Number of Least-Squares Restraints ............       3326 Note
PLAT883_ALERT_1_G No Info/Value for _atom_sites_solution_primary .     Please Do !
PLAT933_ALERT_2_G Number of OMIT Records in Embedded .res File ...          6 Note
PLAT941_ALERT_3_G Average HKL Measurement Multiplicity ..........        2.7 Low
```

---

  0 **ALERT level A** = Most likely a serious problem - resolve or explain
  1 **ALERT level B** = A potentially serious problem, consider carefully
 70 **ALERT level C** = Check. Ensure it is not caused by an omission or oversight
 77 **ALERT level G** = General information/check it is not something unexpected

  3 ALERT type 1 CIF construction/syntax error, inconsistent or missing data
 83 ALERT type 2 Indicator that the structure model may be wrong or deficient
  3 ALERT type 3 Indicator that the structure quality may be low
 57 ALERT type 4 Improvement, methodology, query or suggestion
  2 ALERT type 5 Informative message, check

It is advisable to attempt to resolve as many as possible of the alerts in all categories. Often the minor alerts point to easily fixed oversights, errors and omissions in your CIF or refinement strategy, so attention to these fine details can be worthwhile. In order to resolve some of the more serious problems it may be necessary to carry out additional measurements or structure refinements. However, the purpose of your study may justify the reported deviations and the more serious of these should normally be commented upon in the discussion or experimental section of a paper or in the "special_details" fields of the CIF. checkCIF was carefully designed to identify outliers and unusual parameters, but every test has its limitations and alerts that are not important in a particular case may appear. Conversely, the absence of alerts does not guarantee there are no aspects of the results needing attention. It is up to the individual to critically assess their own results and, if necessary, seek expert advice.

Publication of your CIF in IUCr journals

A basic structural check has been run on your CIF. These basic checks will be run on all CIFs submitted for publication in IUCr journals (*Acta Crystallographica*, *Journal of Applied Crystallography*, *Journal of Synchrotron Radiation*); however, if you intend to submit to *Acta Crystallographica Section C* or *E* or *IUCrData*, you should make sure that full publication checks are run on the final version of your CIF prior to submission.

Publication of your CIF in other journals

Please refer to the *Notes for Authors* of the relevant journal for any special instructions relating to CIF submission.

PLATON version of 13/07/2021; check.def file version of 13/07/2021

Datablock au13 - ellipsoid plot

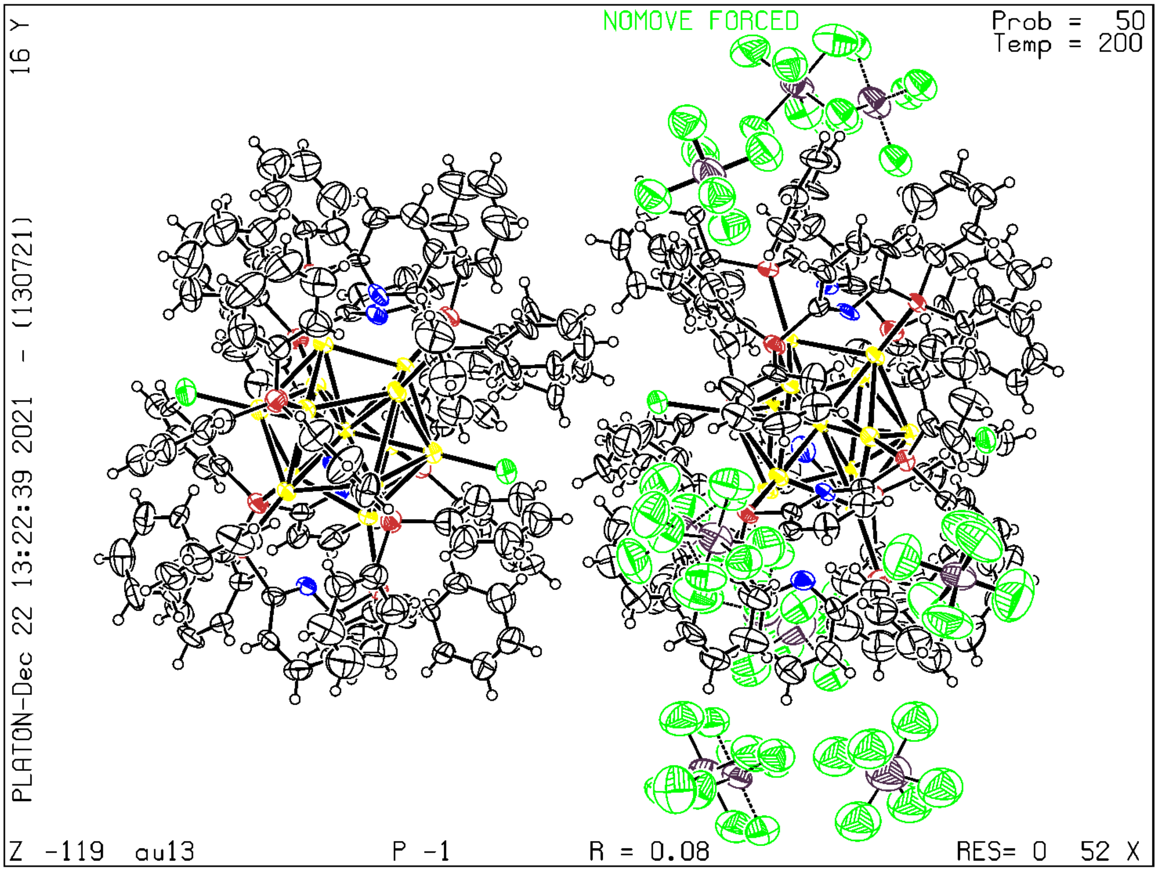

Supplement: nwae174_Supplemental_Files [file nwae174_supplemental_files.zip › Au13-checkcif.pdf]
