# Supplementary material for: Filling the gaps in icosahedral superatomic metal clusters
Source: Natl Sci Rev. 2024 May 28;11(7):nwae174. doi: 10.1093/nsr/nwae174 (PMC11182670; doi:10.1093/nsr/nwae174)

checkCIF/PLATON report

Structure factors have been supplied for datablock(s) auag12

THIS REPORT IS FOR GUIDANCE ONLY. IF USED AS PART OF A REVIEW PROCEDURE FOR PUBLICATION, IT SHOULD NOT REPLACE THE EXPERTISE OF AN EXPERIENCED CRYSTALLOGRAPHIC REFEREE.

No syntax errors found. CIF dictionary Interpreting this report

Datablock: auag12

Bond precision: C-C = 0.0155 A Wavelength=1.54184

Cell:	a=19.1537(1)	b=20.2273(1)	c=22.8288(1)
	alpha=97.233(1)	beta=99.481(1)	gamma=109.458(1)
Temperature:	200 K		

	Calculated	Reported
Volume	8068.56(10)	8068.55(9)
Space group	P -1	P -1
Hall group	-P 1	-P 1
Moiety formula	2(C145 H115 Ag12 Au Cl2 N5 P10), 2(F3.70 Sb0.54), 4(F6 Sb), 2(F	C145 H115 Ag12 Au Cl2 N5 P10, F3.7 Sb0.54, F2.3 Sb0.46, 2(F6 Sb
Sum formula	C293 H236 Ag24 Au2 Cl10 F36 N10 P20 Sb6 [+ solvent]	C146.50 H118 Ag12 Au Cl5 F18 N5 P10 Sb3
Mr	9268.21	4634.06
Dx,g cm-3	1.908	1.907
Z	1	2
Mu (mm-1)	19.232	19.232
F000	4450.0	4450.0
F000’	4464.65	
h,k,lmax	23,25,28	23,25,28
Nref	32613	31503
Tmin,Tmax	0.457,0.562	0.423,1.000
Tmin’	0.274	

Correction method= # Reported T Limits: Tmin=0.423 Tmax=1.000
AbsCorr = MULTI-SCAN

Data completeness= 0.966 Theta(max)= 73.721

R(reflections)= 0.0480(26149)   wR2(reflections)= 0.1257(31503)

S = 1.024   Npar= 2010

The following ALERTS were generated. Each ALERT has the format
test-name_ALERT_alert-type_alert-level.
Click on the hyperlinks for more details of the test.

Alert level C

```
PLAT220_ALERT_2_C NonSolvent   Resd 1  C   Ueq(max)/Ueq(min) Range        4.2 Ratio
PLAT234_ALERT_4_C Large Hirshfeld Difference P10      --C1A      .       0.16 Ang.
PLAT234_ALERT_4_C Large Hirshfeld Difference C3       --C4       .       0.20 Ang.
PLAT234_ALERT_4_C Large Hirshfeld Difference C98      --C99      .       0.17 Ang.
PLAT234_ALERT_4_C Large Hirshfeld Difference C106     --C107     .       0.17 Ang.
PLAT234_ALERT_4_C Large Hirshfeld Difference C127     --C128     .       0.20 Ang.
PLAT241_ALERT_2_C High   'MainMol' Ueq as Compared to Neighbors of        C3 Check
PLAT241_ALERT_2_C High   'MainMol' Ueq as Compared to Neighbors of       C26 Check
PLAT241_ALERT_2_C High   'MainMol' Ueq as Compared to Neighbors of      C131 Check
PLAT244_ALERT_4_C Low    'Solvent' Ueq as Compared to Neighbors of       Sb3 Check
PLAT244_ALERT_4_C Low    'Solvent' Ueq as Compared to Neighbors of       Sb4 Check
PLAT250_ALERT_2_C Large U3/U1 Ratio for Average U(i,j) Tensor ....       2.5 Note
PLAT260_ALERT_2_C Large Average Ueq of Residue Including       Sb1      0.143 Check
PLAT260_ALERT_2_C Large Average Ueq of Residue Including       Sb3      0.112 Check
PLAT260_ALERT_2_C Large Average Ueq of Residue Including       Sb4      0.105 Check
PLAT260_ALERT_2_C Large Average Ueq of Residue Including       Sb2      0.130 Check
PLAT260_ALERT_2_C Large Average Ueq of Residue Including       Cl3      0.184 Check
PLAT260_ALERT_2_C Large Average Ueq of Residue Including       Cl5      0.195 Check
PLAT260_ALERT_2_C Large Average Ueq of Residue Including       Cl7      0.225 Check
PLAT342_ALERT_3_C Low Bond Precision on  C-C Bonds ...............    0.01548 Ang.
PLAT906_ALERT_3_C Large K Value in the Analysis of Variance ......      3.501 Check
PLAT910_ALERT_3_C Missing # of FCF Reflection(s) Below Theta(Min).         5 Note
PLAT911_ALERT_3_C Missing FCF Refl Between Thmin & STh/L=    0.600       240 Report
PLAT971_ALERT_2_C Check Calcd Resid. Dens.  1.04Ang From Cl2            1.68 eA-3
PLAT972_ALERT_2_C Check Calcd Resid. Dens.  0.79Ang From Au1           -2.38 eA-3
PLAT972_ALERT_2_C Check Calcd Resid. Dens.  0.80Ang From Au1           -1.83 eA-3
```

Alert level G

```
FORMU01_ALERT_1_G  There is a discrepancy between the atom counts in the
            _chemical_formula_sum and _chemical_formula_moiety. This is
            usually due to the moiety formula being in the wrong format.
            Atom count from _chemical_formula_sum:   C146.5 H118 Ag12 Au1 Cl5 F18
            Atom count from _chemical_formula_moiety:C146.5 H118 Ag12 Au1 Cl2 F18
PLAT002_ALERT_2_G Number of Distance or Angle Restraints on AtSite        35 Note
PLAT003_ALERT_2_G Number of Uiso or Uij Restrained non-H Atoms ...        53 Report
PLAT045_ALERT_1_G Calculated and Reported Z Differ by a Factor ...     0.500 Check
PLAT083_ALERT_2_G SHELXL Second Parameter in WGHT  Unusually Large     65.77 Why ?
PLAT154_ALERT_1_G The s.u.'s on the Cell Angles are Equal ..(Note)     0.001 Degree
PLAT172_ALERT_4_G The CIF-Embedded .res File Contains DFIX Records         5 Report
PLAT173_ALERT_4_G The CIF-Embedded .res File Contains DANG Records         1 Report
PLAT174_ALERT_4_G The CIF-Embedded .res File Contains FLAT Records         1 Report
PLAT175_ALERT_4_G The CIF-Embedded .res File Contains SAME Records         1 Report
PLAT176_ALERT_4_G The CIF-Embedded .res File Contains SADI Records         1 Report
PLAT177_ALERT_4_G The CIF-Embedded .res File Contains DELU Records         3 Report
```

```
PLAT186_ALERT_4_G The CIF-Embedded .res File Contains ISOR Records          6 Report
PLAT232_ALERT_2_G Hirshfeld Test Diff (M-X)  Ag12     --Cl2      .       17.6 s.u.
PLAT300_ALERT_4_G Atom Site Occupancy of Cl3        Constrained at        0.5 Check
PLAT300_ALERT_4_G Atom Site Occupancy of Cl4        Constrained at        0.5 Check
PLAT300_ALERT_4_G Atom Site Occupancy of C146       Constrained at        0.5 Check
PLAT300_ALERT_4_G Atom Site Occupancy of H14B       Constrained at        0.5 Check
PLAT300_ALERT_4_G Atom Site Occupancy of H14C       Constrained at        0.5 Check
PLAT300_ALERT_4_G Atom Site Occupancy of Cl5        Constrained at        0.5 Check
PLAT300_ALERT_4_G Atom Site Occupancy of Cl6        Constrained at        0.5 Check
PLAT300_ALERT_4_G Atom Site Occupancy of C147       Constrained at        0.5 Check
PLAT300_ALERT_4_G Atom Site Occupancy of H14D       Constrained at        0.5 Check
PLAT300_ALERT_4_G Atom Site Occupancy of H14E       Constrained at        0.5 Check
PLAT300_ALERT_4_G Atom Site Occupancy of Cl7        Constrained at        0.5 Check
PLAT300_ALERT_4_G Atom Site Occupancy of Cl8        Constrained at        0.5 Check
PLAT300_ALERT_4_G Atom Site Occupancy of C148       Constrained at        0.5 Check
PLAT300_ALERT_4_G Atom Site Occupancy of H14F       Constrained at        0.5 Check
PLAT300_ALERT_4_G Atom Site Occupancy of H14G       Constrained at        0.5 Check
PLAT301_ALERT_3_G Main Residue  Disorder .............(Resd  1  )          9% Note
PLAT302_ALERT_4_G Anion/Solvent/Minor-Residue Disorder (Resd  2  )        100% Note
PLAT302_ALERT_4_G Anion/Solvent/Minor-Residue Disorder (Resd  5  )        100% Note
PLAT302_ALERT_4_G Anion/Solvent/Minor-Residue Disorder (Resd  6  )        100% Note
PLAT302_ALERT_4_G Anion/Solvent/Minor-Residue Disorder (Resd  7  )        100% Note
PLAT302_ALERT_4_G Anion/Solvent/Minor-Residue Disorder (Resd  8  )        100% Note
PLAT304_ALERT_4_G Non-Integer Number of Atoms in ..... (Resd  2  )       4.24 Check
PLAT304_ALERT_4_G Non-Integer Number of Atoms in ..... (Resd  5  )       2.76 Check
PLAT304_ALERT_4_G Non-Integer Number of Atoms in ..... (Resd  6  )       2.50 Check
PLAT304_ALERT_4_G Non-Integer Number of Atoms in ..... (Resd  7  )       2.50 Check
PLAT304_ALERT_4_G Non-Integer Number of Atoms in ..... (Resd  8  )       2.50 Check
PLAT432_ALERT_2_G Short Inter X...Y Contact  F2       ..C148     .       2.92 Ang.
                                                   1+x,y,z  =      1_655 Check
PLAT605_ALERT_4_G Largest Solvent Accessible VOID in the Structure        175 A**3
PLAT790_ALERT_4_G Centre of Gravity not Within Unit Cell: Resd.  #          3 Note
              F6 Sb
PLAT790_ALERT_4_G Centre of Gravity not Within Unit Cell: Resd.  #          4 Note
              F6 Sb
PLAT790_ALERT_4_G Centre of Gravity not Within Unit Cell: Resd.  #          6 Note
              C H2 Cl2
PLAT790_ALERT_4_G Centre of Gravity not Within Unit Cell: Resd.  #          7 Note
              C H2 Cl2
PLAT794_ALERT_5_G Tentative Bond Valency for Sb3       (V)       .       5.27 Info
PLAT794_ALERT_5_G Tentative Bond Valency for Sb4       (V)       .       5.20 Info
PLAT860_ALERT_3_G Number of Least-Squares Restraints ............         477 Note
PLAT883_ALERT_1_G No Info/Value for _atom_sites_solution_primary .     Please Do !
PLAT912_ALERT_4_G Missing # of FCF Reflections Above STh/L=  0.600        865 Note
PLAT933_ALERT_2_G Number of HKL-OMIT Records in Embedded .res File         32 Note
PLAT941_ALERT_3_G Average HKL Measurement Multiplicity ..........         2.8 Low
PLAT978_ALERT_2_G Number C-C Bonds with Positive Residual Density.          0 Info
```

---

0 **ALERT level A** = Most likely a serious problem - resolve or explain
0 **ALERT level B** = A potentially serious problem, consider carefully
26 **ALERT level C** = Check. Ensure it is not caused by an omission or oversight
54 **ALERT level G** = General information/check it is not something unexpected

4 ALERT type 1 CIF construction/syntax error, inconsistent or missing data

```
22 ALERT type 2 Indicator that the structure model may be wrong or deficient
 7 ALERT type 3 Indicator that the structure quality may be low
45 ALERT type 4 Improvement, methodology, query or suggestion
 2 ALERT type 5 Informative message, check
```

It is advisable to attempt to resolve as many as possible of the alerts in all categories. Often the minor alerts point to easily fixed oversights, errors and omissions in your CIF or refinement strategy, so attention to these fine details can be worthwhile. In order to resolve some of the more serious problems it may be necessary to carry out additional measurements or structure refinements. However, the purpose of your study may justify the reported deviations and the more serious of these should normally be commented upon in the discussion or experimental section of a paper or in the "special_details" fields of the CIF. checkCIF was carefully designed to identify outliers and unusual parameters, but every test has its limitations and alerts that are not important in a particular case may appear. Conversely, the absence of alerts does not guarantee there are no aspects of the results needing attention. It is up to the individual to critically assess their own results and, if necessary, seek expert advice.

Publication of your CIF in IUCr journals

A basic structural check has been run on your CIF. These basic checks will be run on all CIFs submitted for publication in IUCr journals (*Acta Crystallographica*, *Journal of Applied Crystallography*, *Journal of Synchrotron Radiation*); however, if you intend to submit to *Acta Crystallographica Section C* or *E* or *IUCrData*, you should make sure that full publication checks are run on the final version of your CIF prior to submission.

Publication of your CIF in other journals

Please refer to the *Notes for Authors* of the relevant journal for any special instructions relating to CIF submission.

PLATON version of 09/05/2022; check.def file version of 21/03/2022

Datablock auag12 - ellipsoid plot

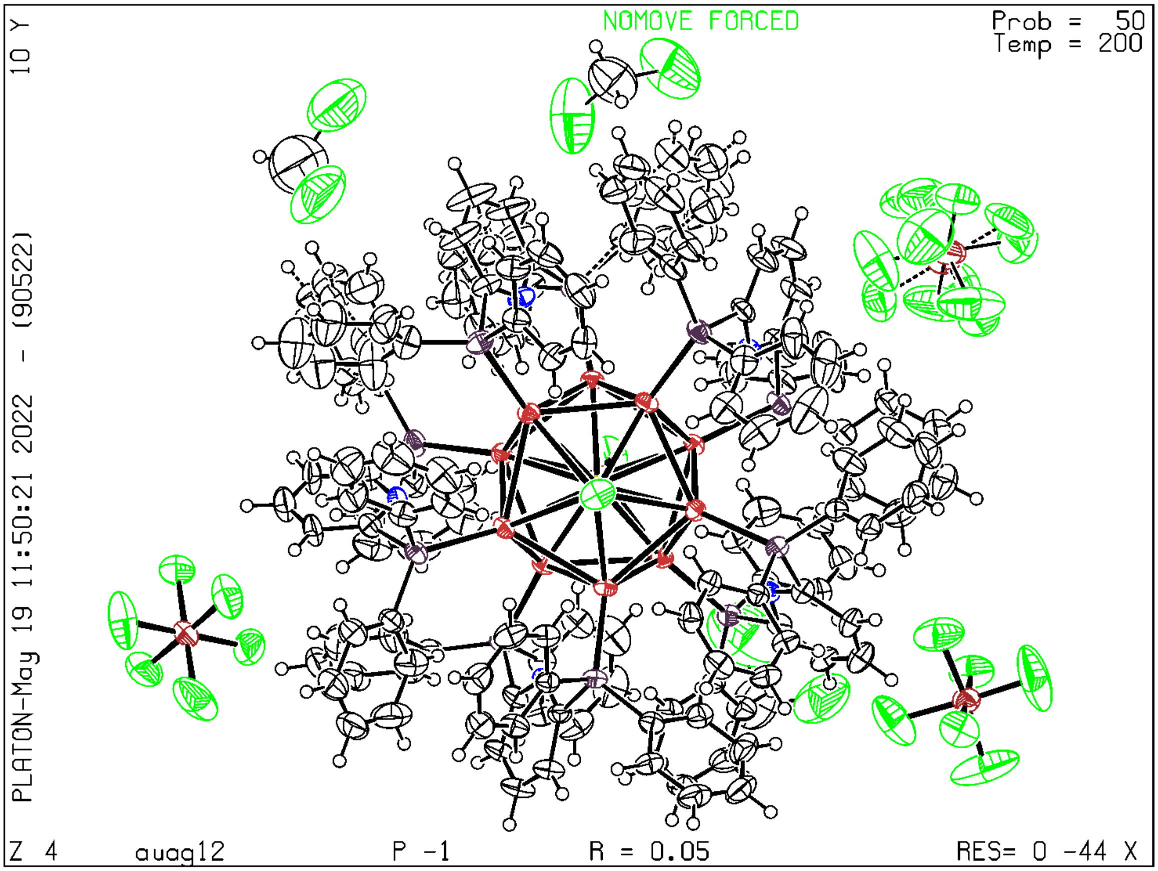

Supplement: nwae174_Supplemental_Files [file nwae174_supplemental_files.zip › AuAg12-checkcif.pdf]
