# Supplementary material for: Filling the gaps in icosahedral superatomic metal clusters
Source: Natl Sci Rev. 2024 May 28;11(7):nwae174. doi: 10.1093/nsr/nwae174 (PMC11182670; doi:10.1093/nsr/nwae174)
Supplement: nwae174_Supplemental_Files [file nwae174_supplemental_files.zip › Supplementary Materials of NSR_MS-2024-171.R2.docx]

**Filling the Gaps in Icosahedral Superatomic Metal Clusters**

Wei-Miao He,^1,†^ Jia-Hua Hu,^1,†^ Yu-Jia Cui,^1^ Jing Li,^1,2, *^ Yu-Bing Si,^1^ Shuai-Bo Wang,^1^ Yu-Jing Zhao,^1^ Zhan Zhou,^1,3, **^ Lu-Fang Ma,^1,3, ***^ Shuang-Quan Zang,^1,4, ****^

^1^College of Chemistry, Zhengzhou University, 450001, Zhengzhou, China

^2^School of Science, Xuchang University, 461000, Xuchang, China

^3^College of Chemistry and Chemical Engineering, Henan Key Laboratory of Function-Oriented Porous Materials, Luoyang Normal University, Luoyang, 471934, China

^4^Lead contact

^*^ Correspondence: lijing42@xcu.edu.cn

^**^Correspondence: zhouzhan@lynu.edu.cn

^***^Correspondence: mazhuxp@126.com

^****^ Correspondence: zangsqzg@zzu.edu.cn

^†^Equally contributed to this work.

**Contents**

**1. Experimental.........................................................................................................S2**

**1.1 Reagents...............................................................................................................S2**

**1.2 Apparatus.............................................................................................................S2**

**1.3 X-ray crystallography............................................................................S3**

**1.4 Calculation procedure.....................................................................................S4**

**1.5 Synthesis.......................................................................................................S5**

**1.6 Cytotoxicity analysis.............................................................................................S7**

**1.7 In the cell imaging experiment............................................................................S7**

**1.8 References..........................................................................................................S8**

**2. Selected spectra and data referred in the paper................................................S9**

**1. Experimental**

**1.1 Reagents**

All chemicals and solvents obtained from suppliers were used without further purification. All solvents were analytical grade reagent. RPMI-1640 and Dulbecco’s phosphate-buffered saline (DPBS; no calcium, no magnesium) were purchased from Gibco™. The Cell Counting Kit-8 (CCK-8) was purchased from Beyotime. mPEG 2000-b-PLGA 10000 (lactide:glycolide = 50:50) (mPEG-PLGA) was obtained from Shanghai Yanyi Biotechnology Co., Ltd (China).

**1.2 Apparatus**

Elemental analyses (EA) were carried out with a Perkin-Elmer 240 elemental analyzer. The high-resolution electrospray ionization time-of-flight mass spectrometry (HRESI-TOF-MS Spectrometry) of **Ag_13_**, **Au*_n_*Ag_13-_*_n,_*** and **Au_13_** were collected on an AB Sciex X500R Q-TOF spectrometer. X-ray photoelectron spectroscopy (XPS) measurements were performed using a K-ALPHA instrument. Inductively Coupled Plasma Optical Emission Spectrometry (ICP-OES) measurements were performed using an Agilent ICPOES730 instrument. Confocal imaging was performed with a Leica TCS SP8 confocal fluorescence microscope. The TEM images were carried out with a FEI Talos F200x instrument. DLS measurements were acquired by a Nano Plus-3 zeta/ General mode. UV-Vis spectra were carried out with a Hitachi UH4150 UV-Visible spectrophotometer. The NIR region luminescence spectra of **Ag_13_**, **Au*_n_*Ag_13-_*_n_*** and **Au_13_** in solid and solution states were measured with an Edinburgh FLS 800 luminescence spectrometer. Luminescence lifetime was measured on a HORIBA FluoroLog-3 fluorescence spectrometer equipped with a 370 nm-laser, operating in time-correlated single-photon counting (TCSPC) mode. The photoluminescent quantum efficiency in solid and solution states was operated using an integrating sphere with excitation at 360 nm on the HAMAMATSU Quantaurus-QY spectrofluorometer.

The transient absorption (TA) spectra were recorded on a commercial pump–probe system (Helios-EOS, Ultrafast Systems LLC) in combination with a femtosecond laser system (Astrella, Coherent). Laser pulses (~800 nm center wavelength, <100 fs duration, 1 kHz repetition rate, ~7 mJ/pulse) were generated by a Ti: Sapphire-based regenerative amplified laser system. The laser pulses were spilt to generate pump and probe beam. The pump pulses at 360 nm were delivered by an optical parametric amplifier which was excited by a portion of 800 nm laser pulses. The pump pulse energy in each measurement was ~25 μJ/cm^2^ at the sample cell. For femtosecond transient absorption (100 fs - 7 ns delay region) measurement, the probe and reference beam were generated by focusing the 800 nm beam (split from the amplifier with a tiny portion) onto a CaF_2_ crystal to generate white-light continuum pulses (350 - 650 nm). The pump−probe delay was controlled by an optical delay line. For nanosecond transient absorption (1 ns - 400 μs delay region) measurement, the pump beam was generated in the same way as the femtosecond TA experiment described above. The probe and reference beam were provided by an additional supercontinuum laser (370-900 nm, ~0.5 ns duration, 2 kHz repetition rate). The pump−probe delay time was controlled electronically in nanosecond TA measurement. In femtosecond and nanosecond TA spectrometers, the intensities of the probe and reference beam were detected on a pair of linear array detectors to obtain the TA signal. The global and target analyses were performed on the glotaran software.

**1.3 X-ray crystallography.**

SCXRD measurements were performed using a Rigaku XtaLAB Pro diffractometer with Cu-Kα radiation (λ = 1.54184 Å). The structures were solved using intrinsic phasing methods (SHELXT-2015) and refined by full-matrix least squares on F^2^ using OLEX2, which utilizes the SHELXL-2018/3 module ^[1]^. All hydrogen atoms were placed in their calculated positions with idealized geometries, and they possessed fixed isotropic displacement parameters.

Appropriate restraints and/or constraints were applied to the geometry, and the atomic displacement parameters of the atoms in the cluster were determined. All non-H atoms were located in the electron density and refined with anisotropic thermal parameters. Due disorder of the partial benzene ring of the P ligands, FLAT restraints were applied to keep the planarity of the six C atoms on the benzene ring. DFIX restraints were applied to keep the distance of C‒C (ca.1.39 Å) of the benzene ring in a reasonable range. AFIX 66 restraints were applied to keep the standard six-membered ring configuration of the phenyl group in the ligands; Due to the disorder of SbF_6_^‒^, SAME/SADI restraints were applied to keep similar configurations. DFIX restraints were applied to keep the distance of Sb‒F (ca.1.85 Å) in a reasonable range; Besides, DFIX restraints were applied to keep the distances of Cl‒C (ca. 1.75 Å) of the CH_2_Cl_2_ molecules in a reasonable range. ISOR, DELU, and SIMU restraints were used for some atoms with large thermal motion. All structures were examined using the Addsym subroutine of PLATON^[2]^ to ensure that no additional symmetry could be applied to the models. A solvent mask has been used due to the severe disorder of free solvent molecules around the cluster and diffract weakly. The crystal structures are visualized by DIAMOND 3.2^[3]^.

Detailed information with respect to the X-ray crystal data, intensity collection procedure, and refinement results for the entire cluster compounds are summarized in Table S13.

**1.4 Calculation procedure.**

The density functional theory (DFT) and time-dependent density functional theory (TD-DFT) calculations were performed with Gaussian 16.^[4]^ **Ag_13_**, **Au_1_Ag_12_**, and **Au_13_** structures were chosen as initial guesses for ground state optimization. The theoretical UV-Vis spectra of **Ag_13_**, **Au_1_Ag_12_**, and **Au_13_** were calculated at the optimized ground-state (S_0_) geometries using TD-DFT under PBE0 functional. All calculations were performed using 6-31g* basis set for H, C, N, Cl, and P atoms^[5,6]^ and Lanl2DZ effective core potentials for Ag and Au atoms. ^[7-9]^

Beginning with the **Ag_13_** cluster, the formation process of **Au_1_Ag_12_** and **Au_3_Ag_10_** clusters was studied, more detail in **Figure S28-29**. The low-lying singlet excited state (S*_n_*, *n* = 1, 2) and triplet excited states (T*_m_*_,_ *m* = 1, 2) of **Au*_n_*Ag_13-_*_n_*** (*n* = 0, 1, 2, 3, 13) are optimized by the Gaussian 16^[4]^ TD-DFT method. The excited-state gradients are calculated to optimize the excited-state geometry. All cluster structures are optimized in the gas phase. The BP86 functional and the 6-31g* basis set for C, H, N, P, and Cl atoms and the LANL2DZ basis for the Ag and Au atoms were adopted. Well-converged geometries are obtained by tightening the energy and gradient convergence criteria to 1×10^−6^ Hartree and 1×10^−3^ Hartree/Å, respectively.

The hole and electron pair distribution analyses were performed using the Multiwfn 3.4. ^[10]^ The input wave function for the analysis of the hole and electron pair distribution of **Ag_13_** and **Au_13_** was calculated using the Gaussian 16 program^[4]^. The PBE0 functional and the 6-31g* basis set for C, H, N, P, and Cl atoms and the LANL2DZ basis for the Ag and Au atoms were adopted. The degree of overlap between holes and electrons is studied based on the calculation of the *S_r_* index and the distance between the hole and electron center of mass (*D*) using the Multiwfn 3.4.^[10]^ The *S_r_* index is defined as the full space integration of a function ($S_{r}\left( r \right)$) describing the overlap between electron and hole distributions, formulated as $S_{r}\left( r \right) index=\int\sqrt{\rho^{hole}(r)\rho^{electron}(r)}dr$, where $\rho^{hole}(r)$ and $\rho^{electron}$ are the hole and electron distribution. *D* index is the distance between a hole and an electron center of mass, formulated as $D index=\sqrt{({D_{x})}^{2}+({D_{y})}^{2}+({D_{z})}^{2}}$.

The spin-orbit coupling matrix elements (SOCME) were calculated by ORCA 5.0.0 software package ^[11]^ based on the PBE0 functional and the DKH-def2-TZVP(-f) basis set (SARC-DKH-TZVP for Au and Ag atoms). For the S*_n_*→T*_m_* ISC process, the SOCME is calculated based on the optimized S*_n_* geometry. For the T_1_→S_0_ ISC process, the SOCME is calculated based on the optimized T_1_ geometry. The contributions of the three degenerate triplet states (T*_m_*_,x_, T*_m_*_,y_, and T*_m_*_,z_) were taken into account by calculating the root sum square of the real and imaginary parts (Re and Im) of the matrix elements, as expressed by the following equation:

$$\left\langle S_{n}\left| Ĥsoc \right|T_{m} \right\rangle=\sqrt{\left| \left\langle S_{n}\left| Ĥsoc \right|T_{m} \right\rangle\right|^{2}}=\sqrt{\underset{J=x,y,z}{\Sigma}\left\langle S_{n}\left| Ĥsoc \right|{T_{m}}^{J} \right\rangle\left\langle S_{n}\left| Ĥsoc \right|{T_{m}}^{J} \right\rangle^{*}}$$

**1.5 Synthesis**

**General procedures:**

**Caution!** Silver ethynides are potentially explosive and should be used with great care in small amounts. Hydrochloric acid is harmful to the human body, please use it with care in the fume hood.

**Synthesis of Ag_13_**

50 mg of [*^t^*BuC≡CAg]*_n_* and 100 mg of 2,6-bis(diphenylphosphino)pyridine were added to a mixture of 10 mL of methanol and 10 mL of dichloromethane and stirred vigorously until colorless and clarified. Then dissolve 10 mg (CH_3_)_3_ CNH_2_•BH_3_ in 2 mL methanol solution and add the above solution. It is observed that the colorless solution turns orange rapidly. Then add counterions such as NaSbF_6_, *^t^*BuNBF_4_, NaBPh_4_ (NaBPh_4_ solution will be cloudy, which is clarified by adding CH_2_Cl_2_), and volatilize at room temperature for about three days to obtain red block crystals (Figure S16).

**Synthesis of** **Au*_n_*Ag_13-_*_n_***

The fabrication process of **Au*_n_*Ag_13-_*_n_*** was analogous to that of **Ag_13_**, with the exception of the incorporation of extra gold sources (Au(CH_3_SCH_3_) Cl). The feeding ratio of 10 - 30% **Au*_n_*Ag_13-_*_n_*** is as follows: [*^t^*BuC≡CAg] *_n_* (50 mg), adding 5 mg, 10 mg and 15 mg Au(CH_3_SCH_3_) Cl, respectively. The reaction mixture, exhibiting red luminescence, is evaporated under vacuum to yield an orange-red solid. This solid is subsequently dissolved in dichloromethane and then diffused through ether to yield crystals after approximately two days.

**Synthesis of Au_13_**

0.05 mmol of dpppy and 0.1 mmol of Au(CH_3_SCH_3_) Cl were stirred overnight in 20 mL of dichloromethane protected from light and the solution was removed by spin evaporation under reduced pressure to give a white solid. Adding 150 mg of dpppy -Au to a mixture of 40 mL of ethanol and 20 mL of dichloromethane, followed by 33.5 mg of NaBH_4_, the solution was found to gradually turn black, and after stirring vigorously for 3 h, 0.5 mL of 12 M HCl was added and stirring was continued for 36 h. The solution was found to turn red with red luminescence. The solvent was removed by spin evaporation, and the resulting solid was dissolved in a mixture of dichloromethane and ethanol, then an excess of NaSbF_6_ was added, filtered, and the resulting solid was recrystallized in ethanol, toluene, and dichloromethane (based on Au, Figure S16).

**Synthesis of Au_13_@PLGA**

1 mL of the mixture solution of DMSO/aceton (v/v = 1:1) containing 1 mg of **Au_13_** and 10 mg of mPEG-PLGA was slowly added to deionized (DI) water (9 mL) under high-speed stirring. After stirring overnight, the solution was diluted with DI water and filtered through an ultrafiltration tube for concentration to obtain the product.

**1.6 Cytotoxicity analysis**

HeLa cells were inoculated into 96-well plates at a density of approximately 10^4^ cells per well, and cultured in RPMI-1640 medium supplemented with 10% fetal bovine serum and 1% penicillin-streptomycin at 37℃ in a humidified incubator with 5% CO_2_. After cell adhesion, the medium was replaced with 100 μL of fresh medium containing different concentrations of **Au_13_@PLGA** (0, 1, 3, 5, 8, 10 μM for Au). After incubation for 24 h, CCK-8 reagent was added to the wells and incubated for another 1.5 h according to the manufacturer’s protocol. The absorbance at 450 nm was measured using a multimode plate reader. The relative cell viability (%) was calculated as follows: (A_test_/A_control_) × 100.

**1.7 In the cell imaging experiment**

HeLa cells were cultured on glass-bottomed Petri dishes at an initial density of 2×10^4^ cells/dish, **Au_13_@PLGA** (4 μM) was added for subsequent 2 h incubation. The cells were fixed with paraformaldehyde (4%) for 15 min, and imaged using a Leica TCS SP8 CLSM (excited at 488 nm).

**1.8 References**

1. Sheldrick GM. Crystal structure refinement with SHELXL. *Acta Cryst Sect C* 2015; **71**: 3−8.

2. Spek A L. Structure validation in chemical crystallography. *Acta Cryst Sect D* 2009; **65**: 148−155.

3. Brandenburg K. *Diamond* 2010.

4. Frisch MJ, Trucks GW and Schlegel HB *et al.* *Gaussian 16 Rev C 01*, Wallingford CT 2016.

5. Hariharan PC and Pople JA. *Theoret* *Chimica Acta* 1973; **28**: 213-222.

6. Francl MM, Petro WJ and Hehre WJ *et al.* *J Chem Phys* 1982; **77**: 3654-3665.

7. Hay PJ and Wadt WR. *J Chem Phys* 1985; **82**: 270-283.

8. Hay PJ and Wadt WR. *J Chem Phys* 1985; **82**: 284-298.

9. Hay PJ and Wadt WR. *J Chem Phys* 1985; **82**: 299-310.

10. Lu T and Chen FW. *J Comput Chem*. 2012; **33**: 580-592.

11. Neese F. Software update: The ORCA program system-version 5.0. *Wiley Interdiscip. Rev Comput Mol Sci*.2022; **12**: e1606.

**2.** **Selected spectra and data referred in the paper**


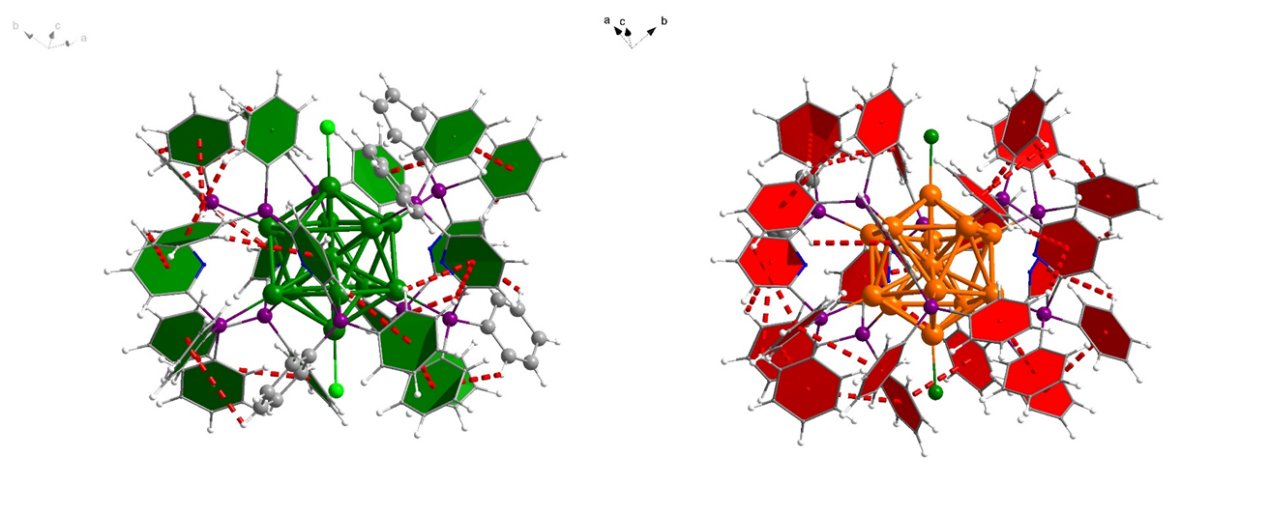


**Figure S1**. Intramolecular weak interactions in **Ag_13_** (left) and **Au_13_** (right) clusters.


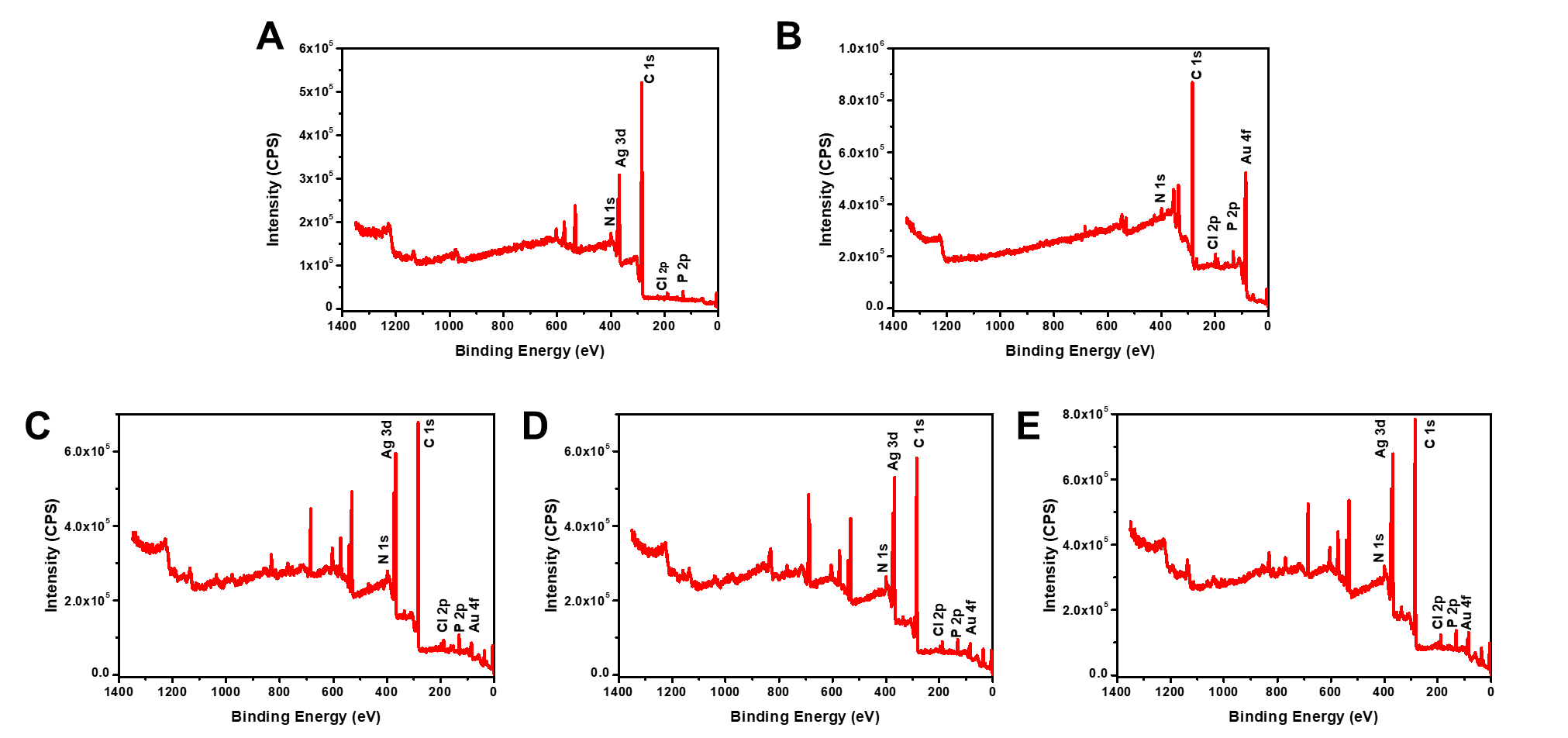


**Figure S2**. Full XPS spectra of (A) **Ag_13_**, (B) **Au_13_**, (C) 10%, (D) 20%, and (E) 30% **Au*_n_*Ag_13-_*_n_***.


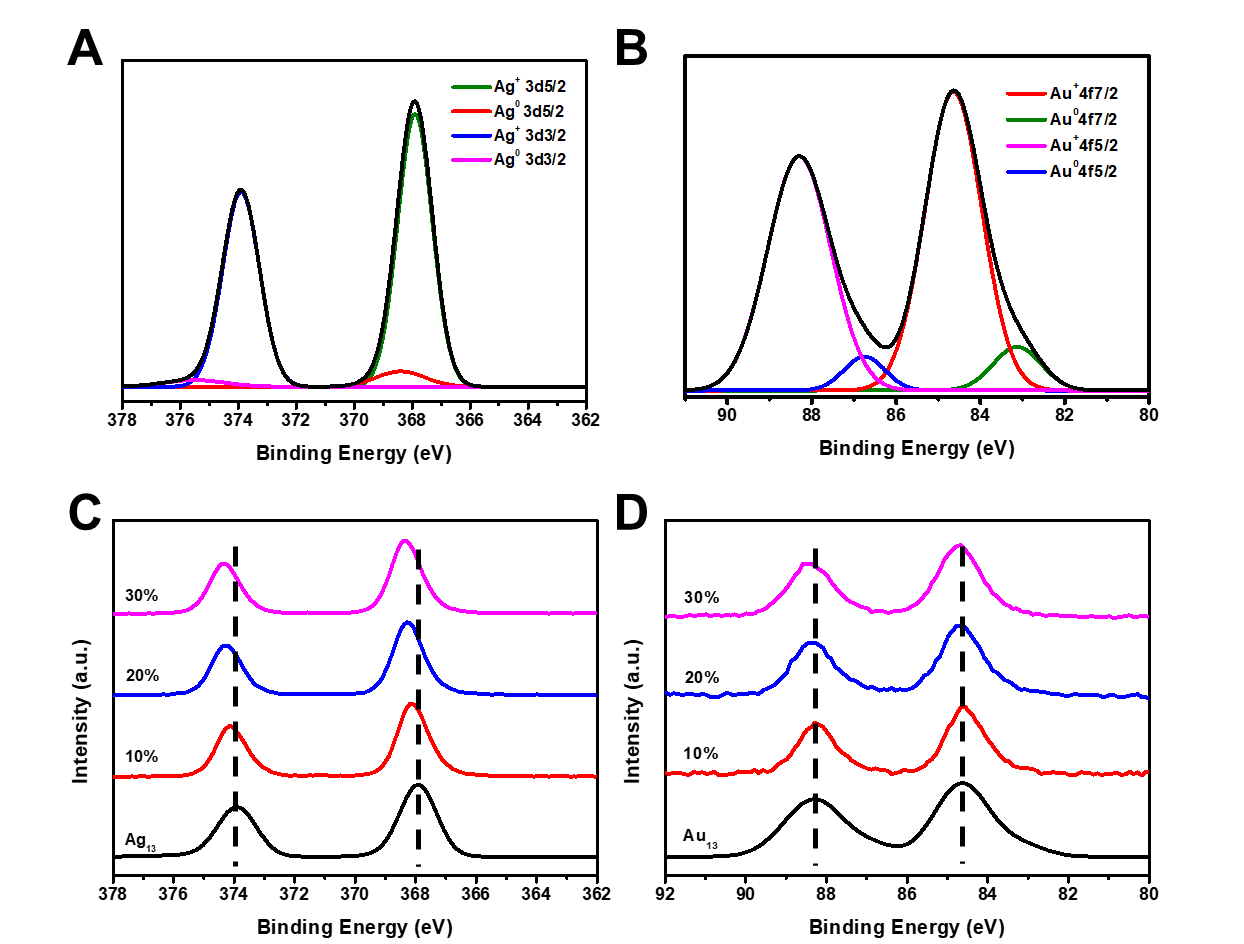


**Figure S3**. High-resolution (A) Ag 3d and (B) Au 4f XPS fitting spectra of **Ag_13_** and **Au_13_**. (C) High- resolution Ag 3d XPS spectra of **Ag_13_**, 10%, 20% and 30% **Au*_n_*Ag_13-_*_n_***. (D) High-resolution Au 4f XPS spectra of **Au_13_**, 10%, 20% and 30% **Au*_n_*Ag_13-_*_n_***.


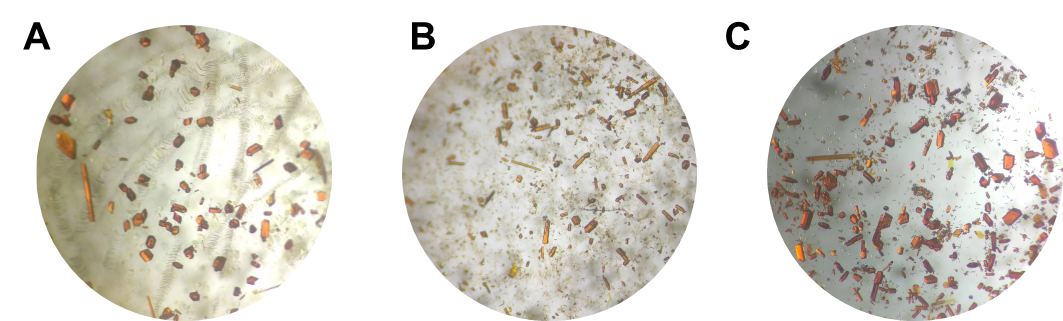


**Figure S4.** The crystal photos of (A) 10%, (B) 20%, and (C) 30% **Au*_n_*Ag_13-_*_n_***.


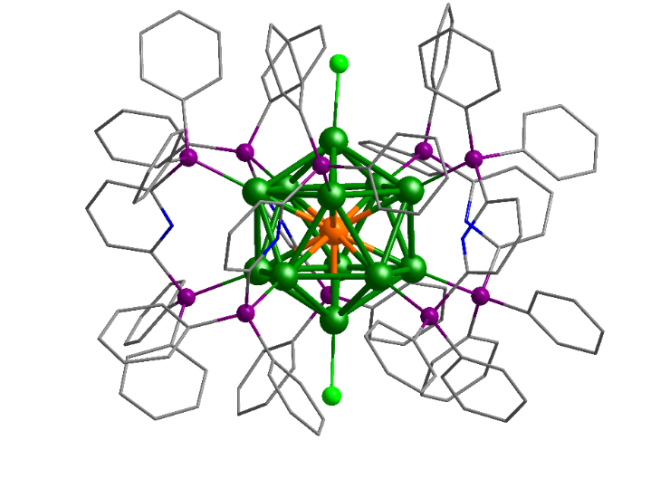


**Figure S5**. The structure of **Au_1_Ag_12_** (omitting the hydrogen atom for clarity).


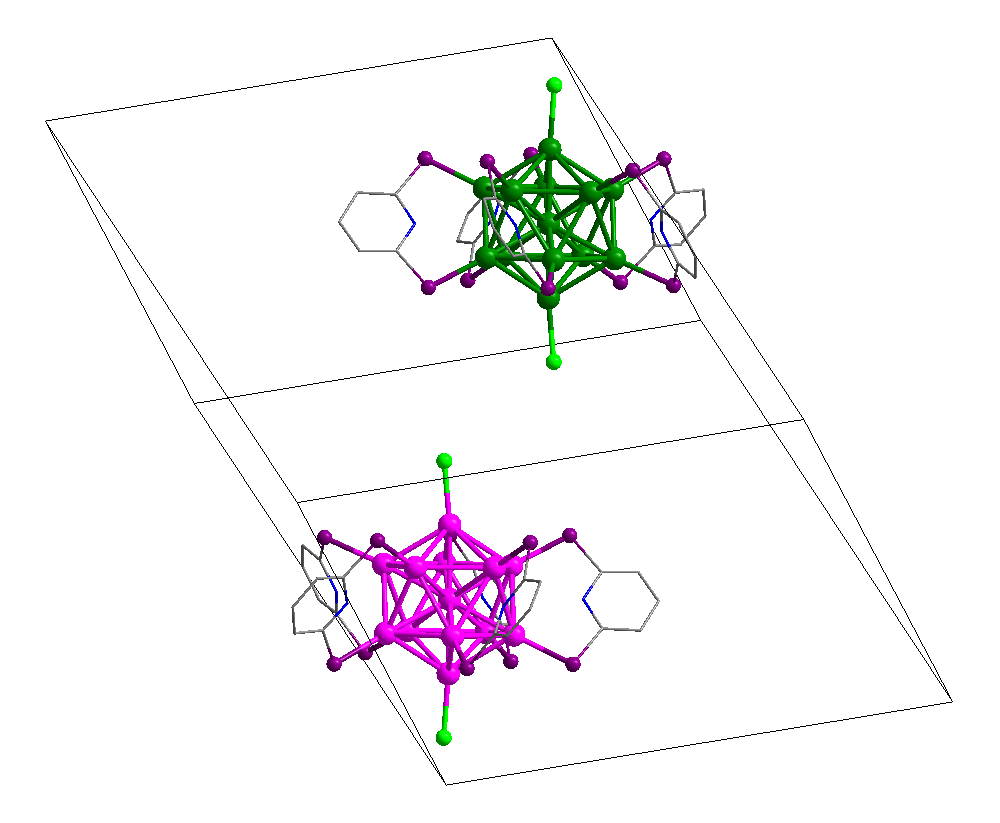


**Figure S6**. A pair of **Ag_13_** in a single cell shows mirror symmetry. Color labels: green and pink, Ag; violet, P; gray, C; blue, N. All benzene rings and H atoms are omitted for clarity.


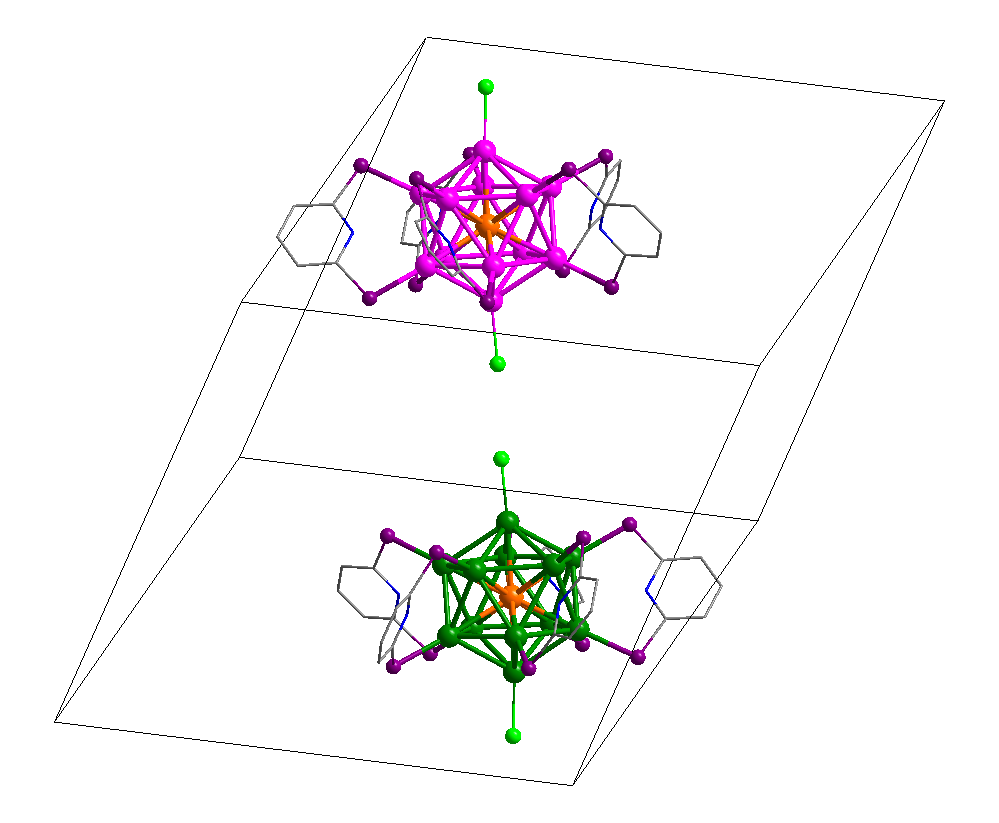


**Figure S7**. A pair of **Au_1_Ag_12_** in a single cell showing mirror symmetry. Color labels: green and pink, Ag. orange, Au; violet, P; gray, C; blue, N. All benzene rings and H atoms are omitted for clarity.


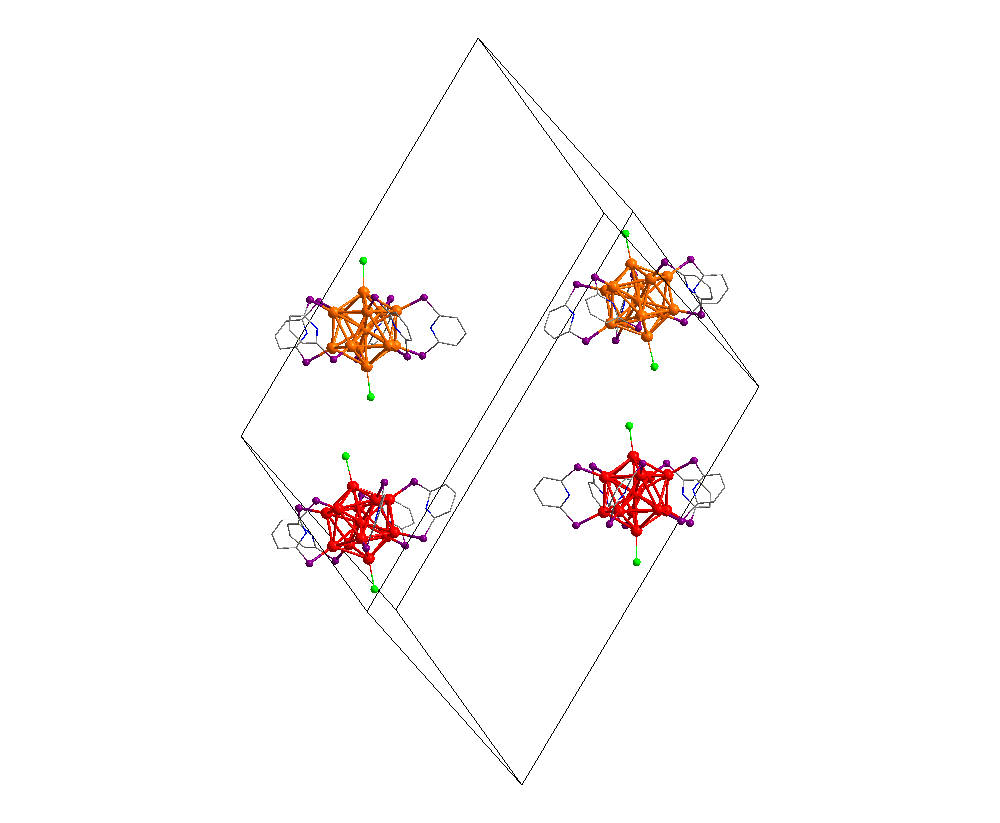


**Figure S8**. Two pairs of **Au_13_** in a single cell show mirror symmetry. Color labels: orange and red, Au; violet, P; gray, C; blue, N. All benzene rings and H atoms are omitted for clarity.

**Figure S9.** The UV-Vis absorption spectra of **Ag_13_** in (A) DMSO, (B) DCM, (C) AC, (D) DMF, (E) DMAc and (F) MeCN solutions at different times.

**Figure S10.** UV-Vis absorption spectra of **Ag_13_**, 10%, 20%, 30%, and **Au_13_** in DMSO.

**Figure S11**. The emission spectra of **Ag_13_** in solid and DMSO.

**Figure S12**. The emission spectra of **Ag_13_** in DMSO (black line) and DMSO + H_2_O (red line), respective at room temperature.


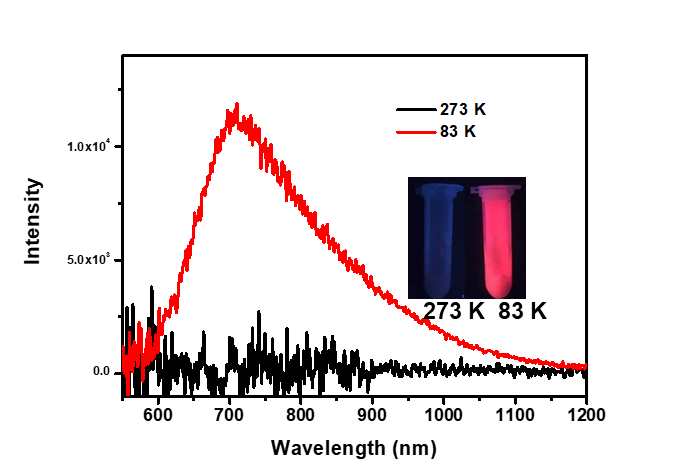


**Figure S13**. The emission spectra of **Ag_13_** in DMSO at 83 K and 273 K.


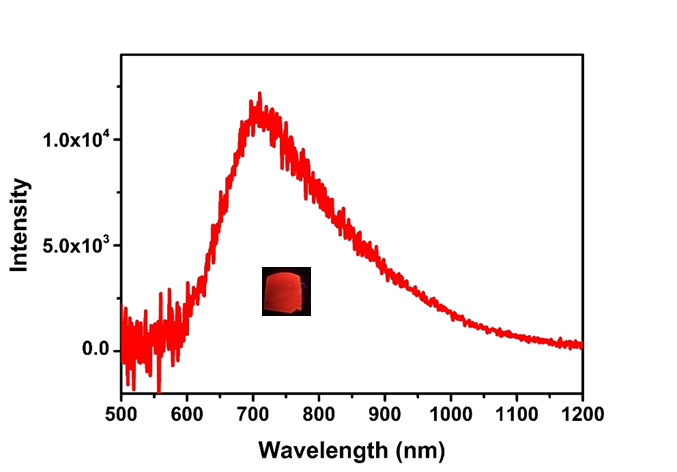


**Figure S14**. The emission spectra of **Ag_13_**@PMMA film at room temperature.

**Figure S15**. The emission spectra of **Au_13_** in solid and DMSO.

**Figure** **S16**. (A) The emission spectra of **Ag_13_** and **Au_13_** in solid state. (B) The images of **Ag_13_** and **Au_13_** under natural and 365 nm light.


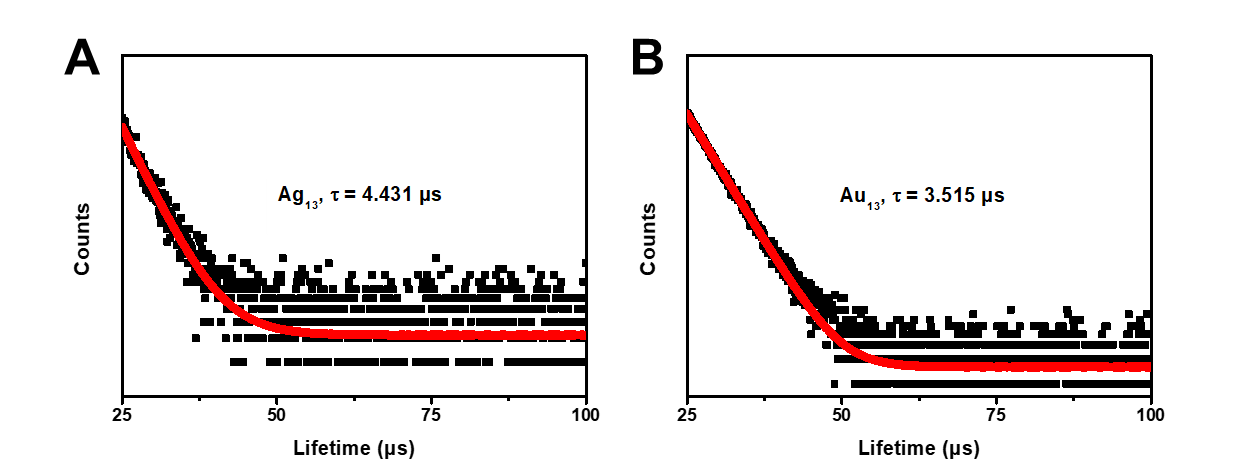


**Figure S17.** Delay lifetime of (A) **Ag_13_** and (B) **Au_13_** solid state at room temperature.

**Figure S18**. The emission spectra of **Ag_13_** and **Au_13_** in DMSO solution.


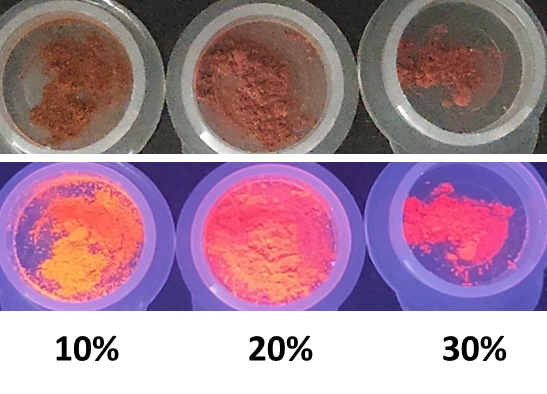


**Figure S19**. The photograph of **Au*_n_*Ag_13-_*_n_*** (10%, 20%, and 30%) under ambient light and UV lamp (365 nm).

**Figure S20**. Emission spectra of 10%, 20%, and 30%.


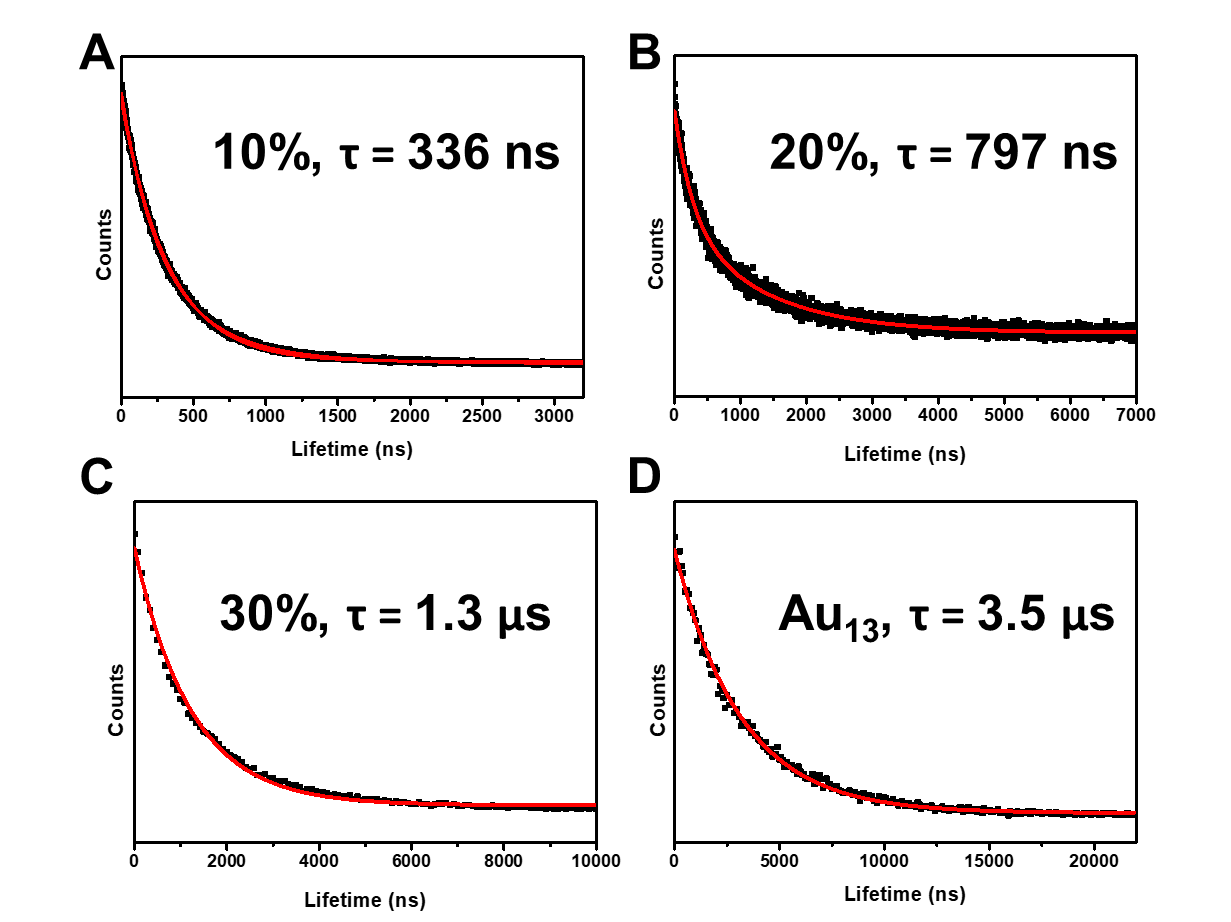


**Figure S21**. The decay time of (A) 10%, (B) 20%, (C) 30%, and (D) **Au_13_** in DMSO solution.


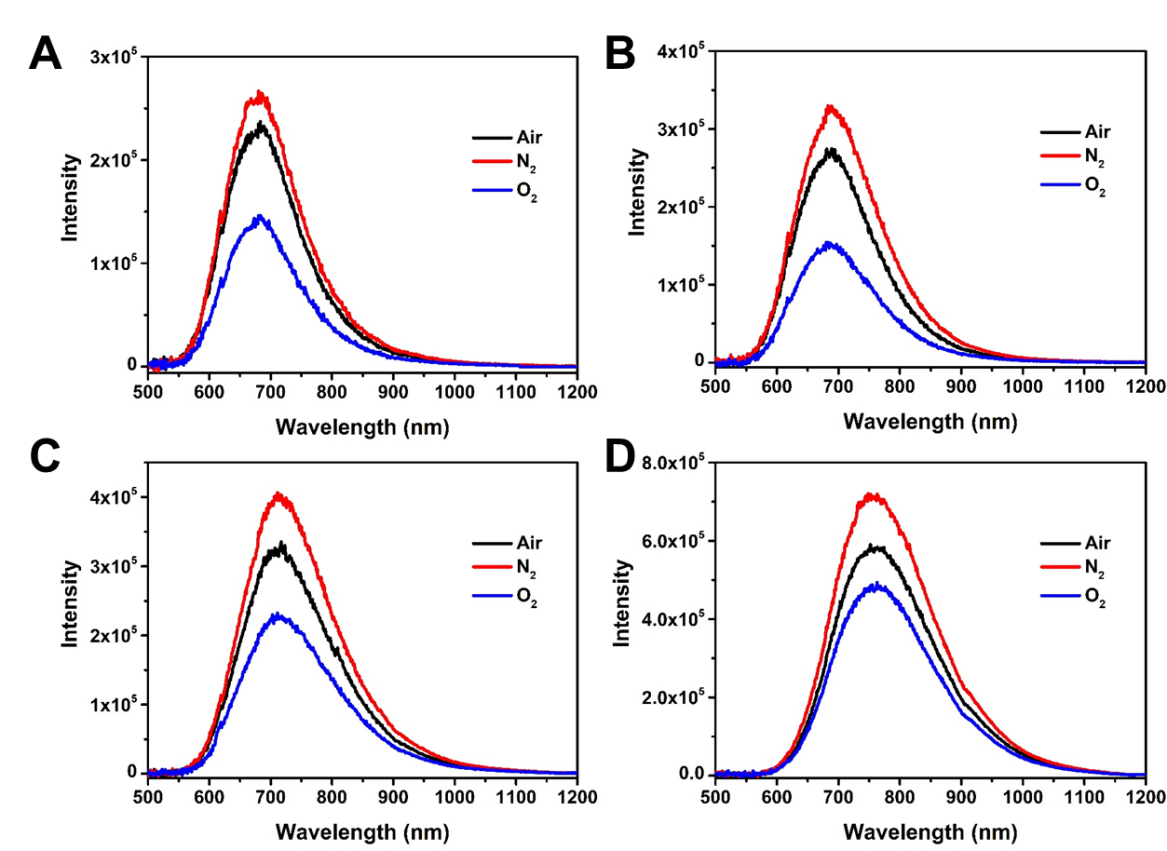


**Figure S22**. Emission spectra of (A) 10%, (B) 20%, (C) 30%, and (D) **Au_13_** in O_2_, N_2_, and Air (in DMSO).

**Figure S23**. Solid emission spectra of (A) **Ag_13_**, (B) **Au_13_**, (C) 10%, (D) 20%, and (E) 30% in O_2_, N_2_, and Air.


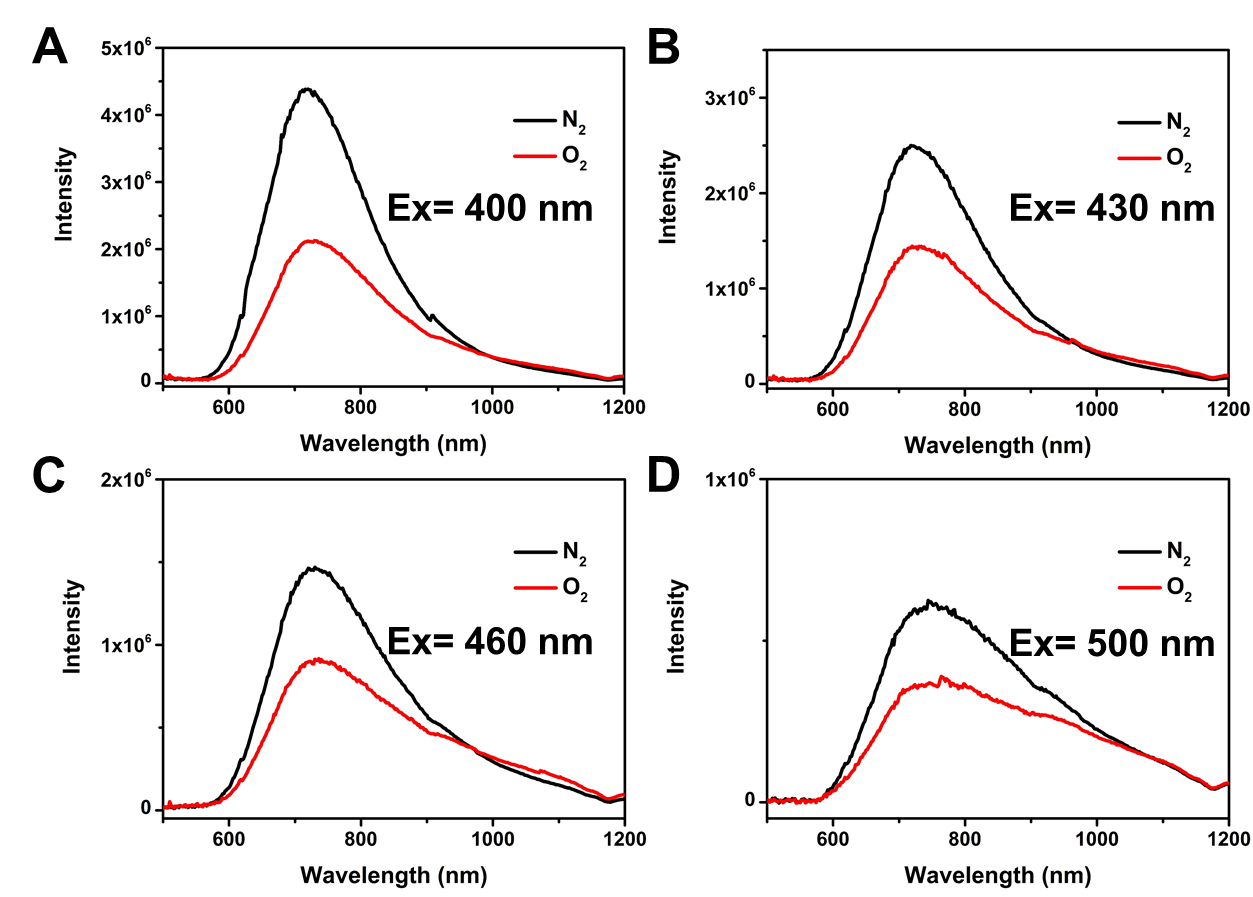


**Figure S24**. Emission spectra of different excitations of 30% under N_2_ and O_2_ (in DMSO).


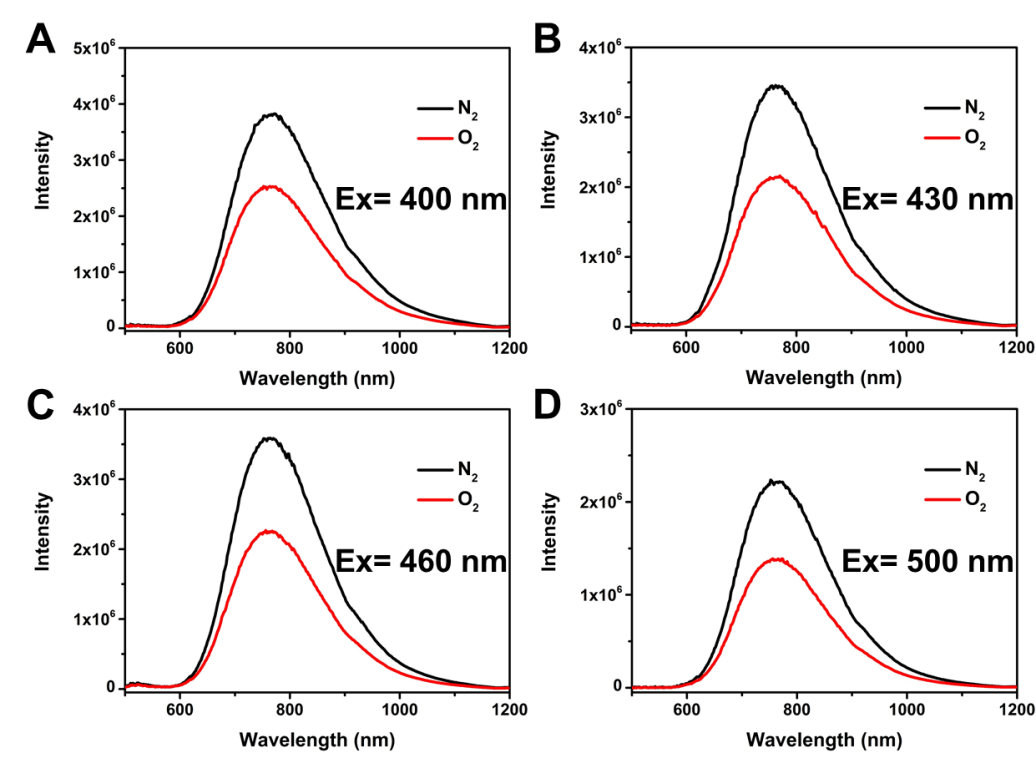


**Figure S25**. Emission spectra of different excitations of **Au_13_** under N_2_ and O_2_ (in DMSO).


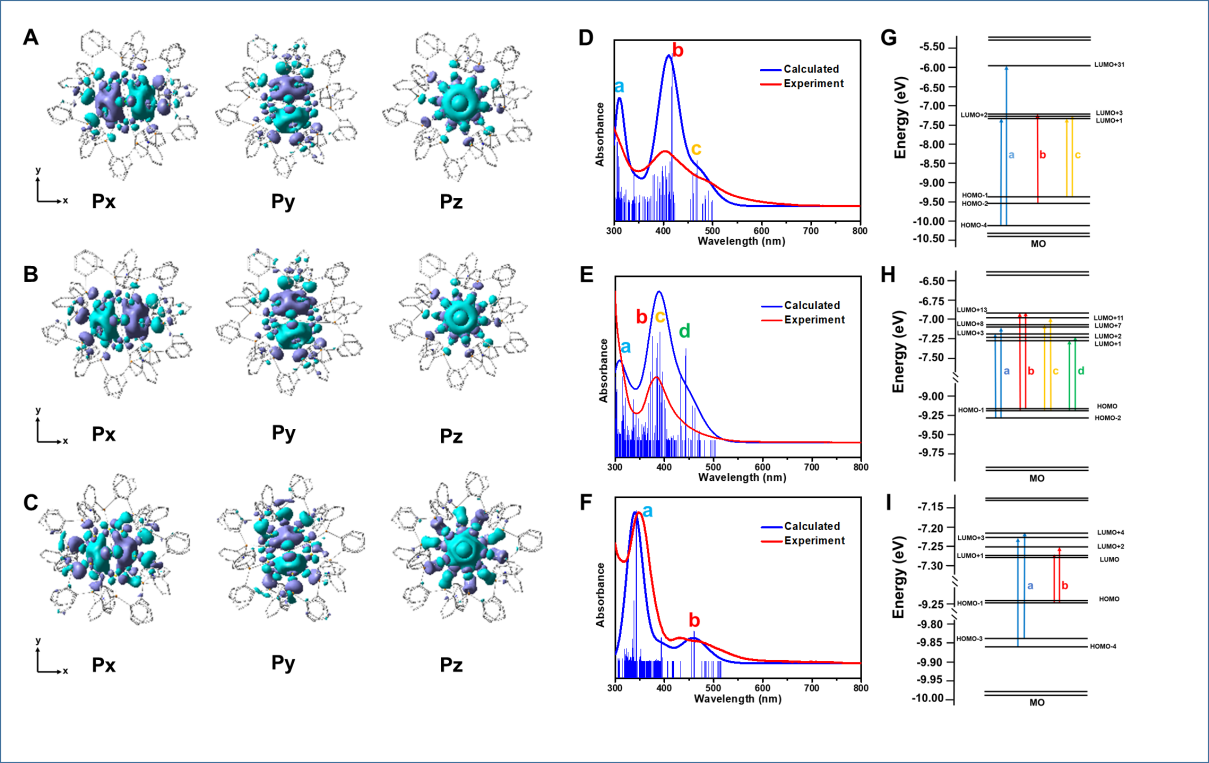


**Figure S26.** The HOMO to HOMO-2 of ground-state (A) **Ag_13_**, (B) **Au_1_Ag_12_,** and (C) **Au_13_** show P-type orbital character. The experimental and theoretical UV/Vis spectra of (D) **Ag_13_**, (E) **Au*_n_*Ag_13-_*_n_*,** and (F) **Au_13_**. The molecular orbitals (MOs) of (G) **Ag_13_**, (H) **Au_1_Ag_12_**, (I) **Au_13_**.


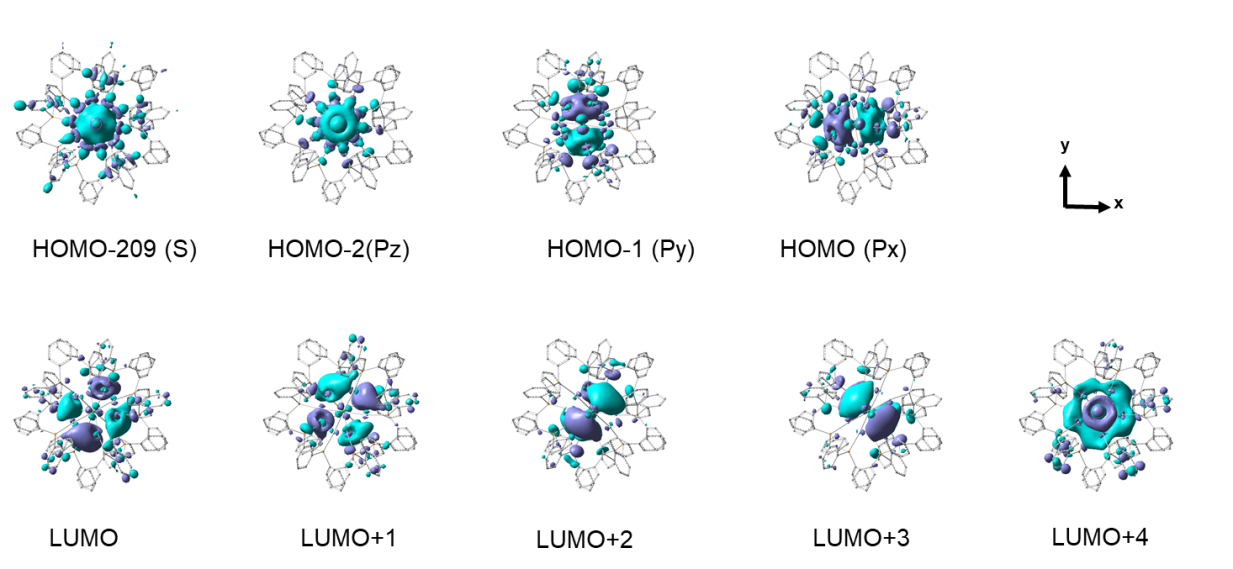


**Figure S27**. Selected molecular orbitals of the **Ag_13_** nanocluster.


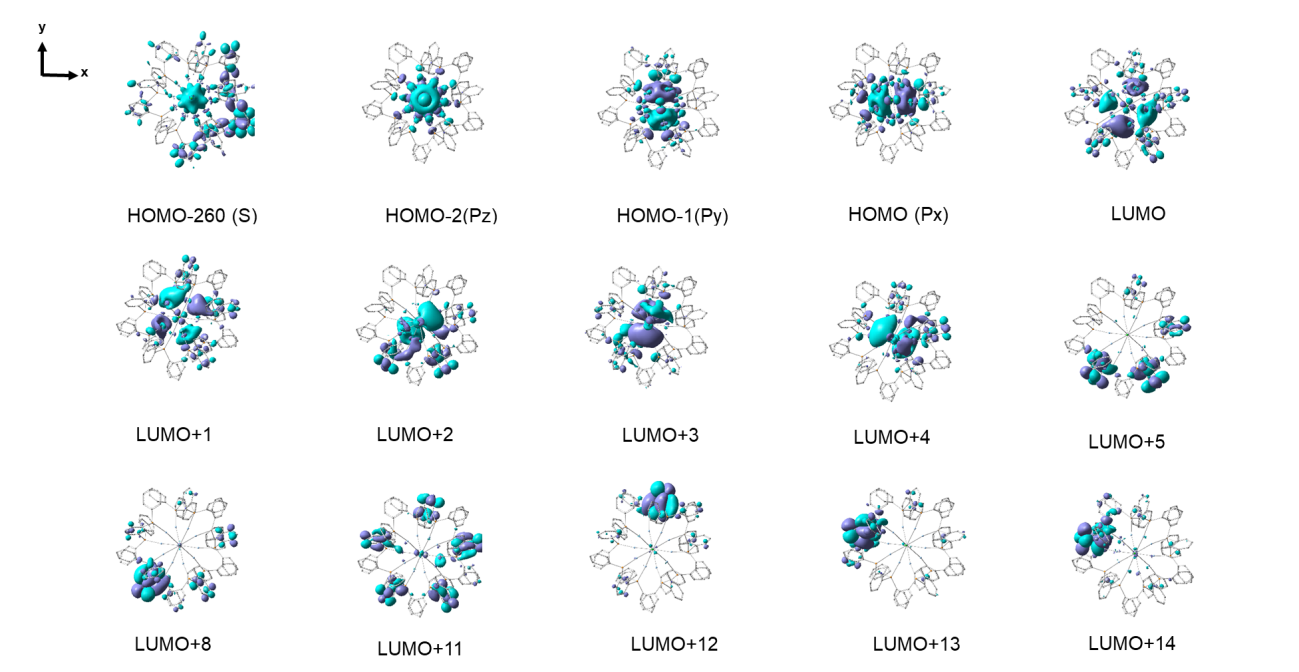


**Figure S28**. Selected molecular orbitals of the **Au_1_Ag_12_** nanocluster.


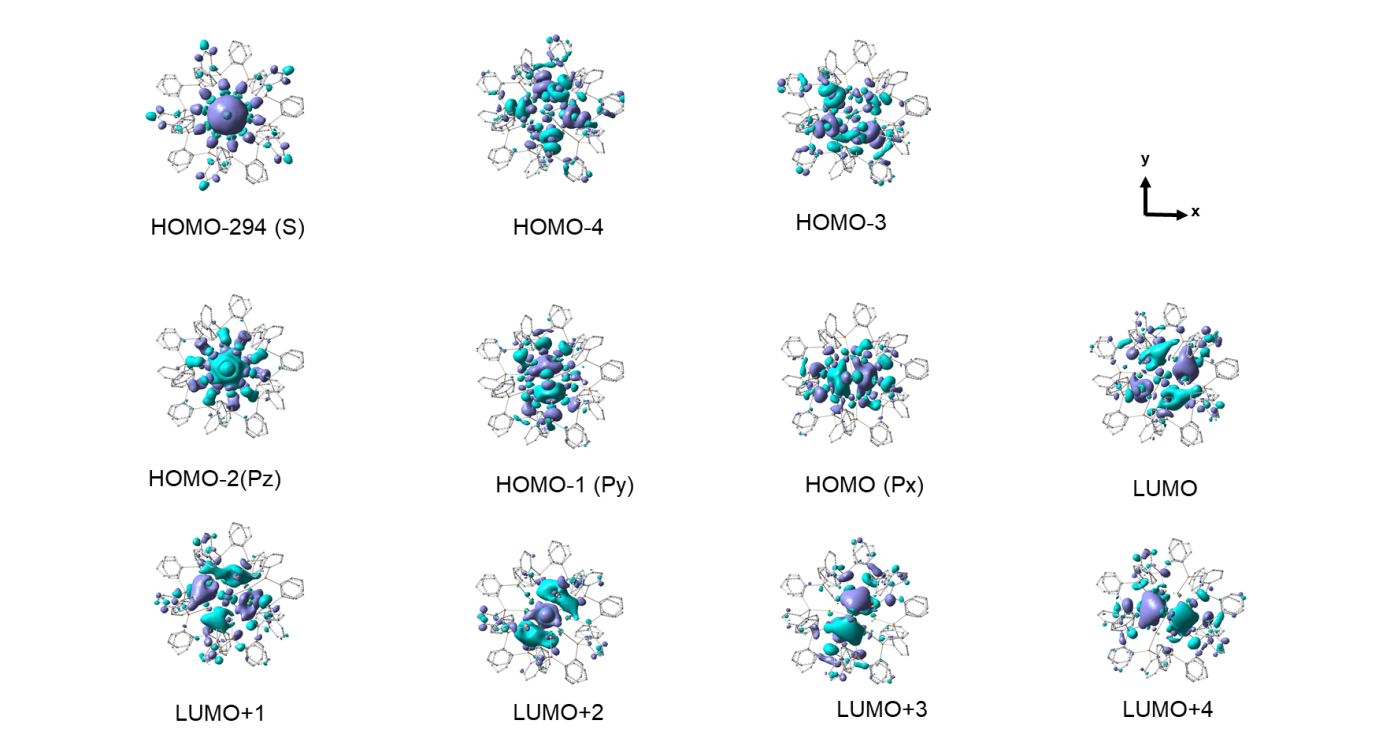


**Figure S29**. Selected molecular orbitals of the **Au_13_** nanocluster.


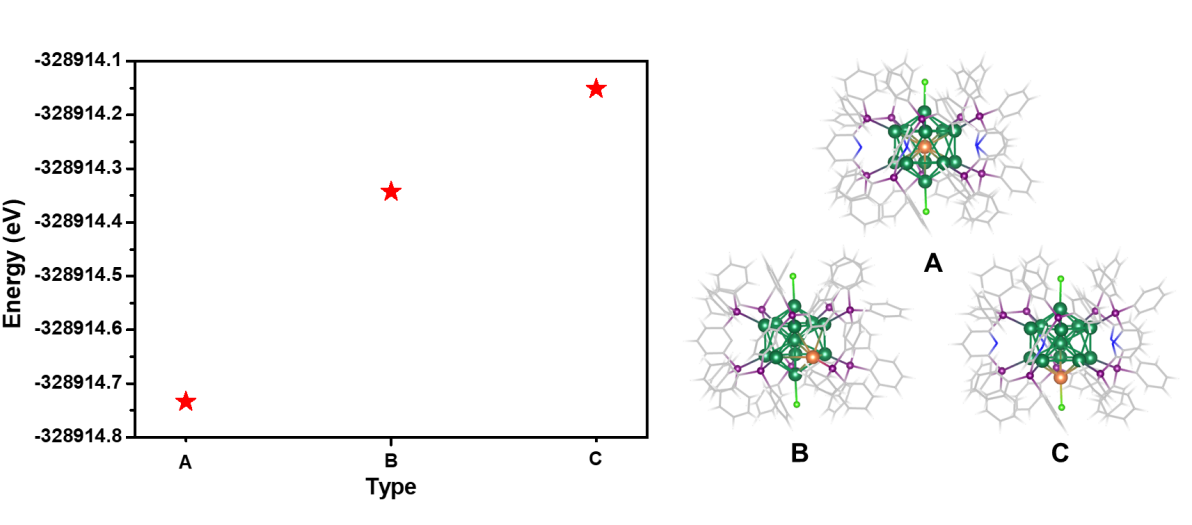


**Figure S30.** Three doping sites and energy sizes of gold atoms in **Au_1_Ag_12_**.


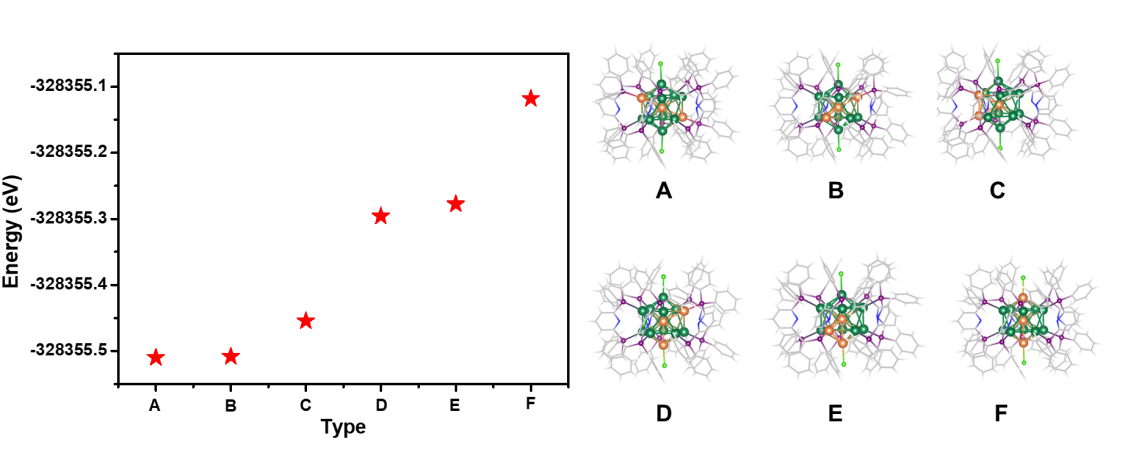


**Figure S31.** Three doping sites and energy sizes of gold atoms in **Au_3_Ag_10_.**

Beginning with the **Ag_13_** cluster, the formation process of **Au_1_Ag_12_** and **Au_3_Ag_10_** clusters was first studied. As shown in **Figure S30**, we considered three substitution positions of a single Au atom. Due to the symmetry of the **Ag_13_** core, there are three equivalent positions corresponding to the A, B, and C isomer clusters. A is more favorable in energy than the other two sets of isomers by 0.39 eV and 0.58 eV, respectively. The theoretically determined stable structure of **Au_1_Ag_12_** was in good agreement with the experimental crystal structure of the Au atom in the center of the **Au_1_Ag_12_** icosahedron, indicating that the theoretical calculations can correctly predict the structural evolution of **Au*_n_*Ag_13-_*_n_*** clusters. Using the same means, the optimal structures of **Au_3_Ag_10_** clusters were determined by comprehensively sampling the substitution positions of Au atoms in the metal core. As shown in **Figure S31**, the most favorable substitution site of the Au atom was determined by two Au atoms into the **Au_1_Ag_12_** (**Au_1_Ag_12_-A**) isomer cluster, in the most stable isomer structure of **Au_3_Ag_10_** two dopant Au atoms adopted a symmetric distribution surrounding a compact icosahedron core, and coordinated with phosphorus atoms.

**Figure S32**. The emission spectra of (A) 10% **Au*_n_*Ag_13-_*_n_***, (B) 20% **Au*_n_*Ag_13-_*_n_***, (C) 30% **Au*_n_*Ag_13-_*_n_*_,_** and (D) **Au_13_** in different solvents.

**Figure S33**. The UV absorption spectra of (E) 10% **Au*_n_*Ag_13-_*_n_***, (F) 20% **Au*_n_*Ag_13-_*_n_***, (G) 30% **Au*_n_*Ag_13-_*_n_***, and (H) **Au_13_** in different solvents.


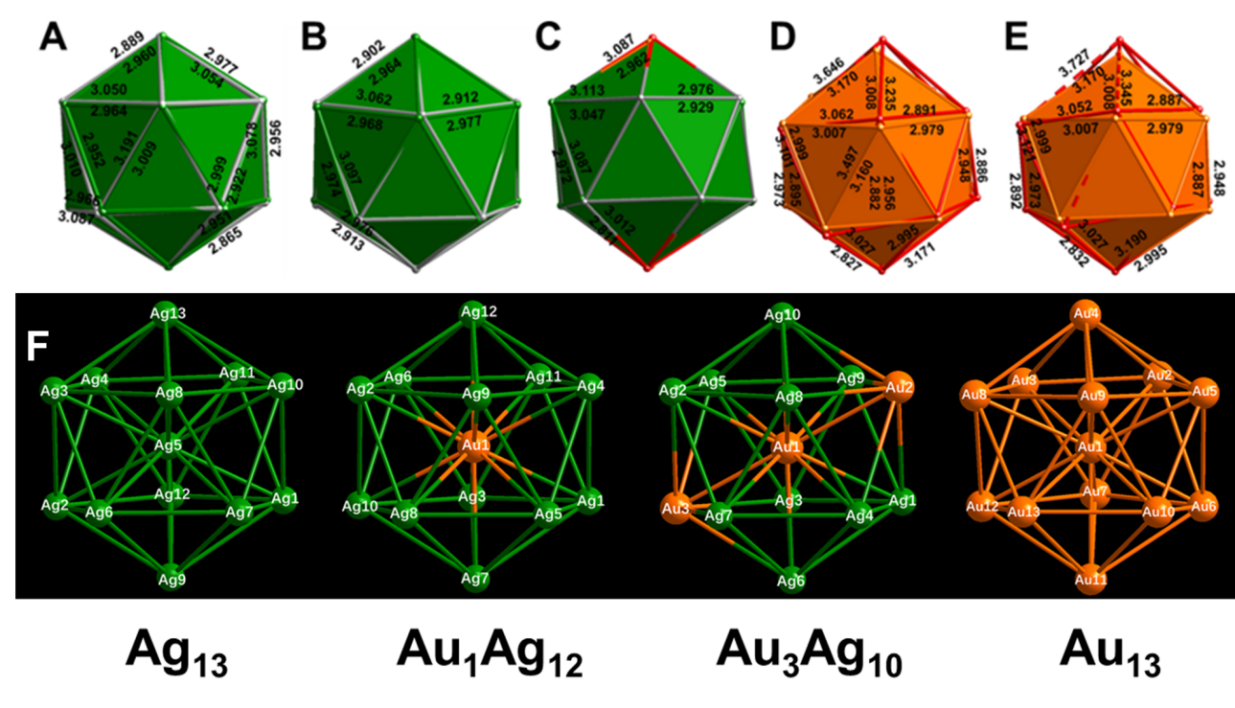


**Figure S34**. S_0_ state and T_1_ state bond length change of (A) **Ag_13_**, (B) **Au_1_Ag_12_**, (C) **Au_3_Ag_10_**, and (D) **Au_13_**. S_0_ state and T_2_ state bond length change of (E) **Au_13_**. (F) Atomic numbers of **Ag_13_**, **Au_1_Ag_12_**, **Au_3_Ag_10_**, and **Au_13_**.


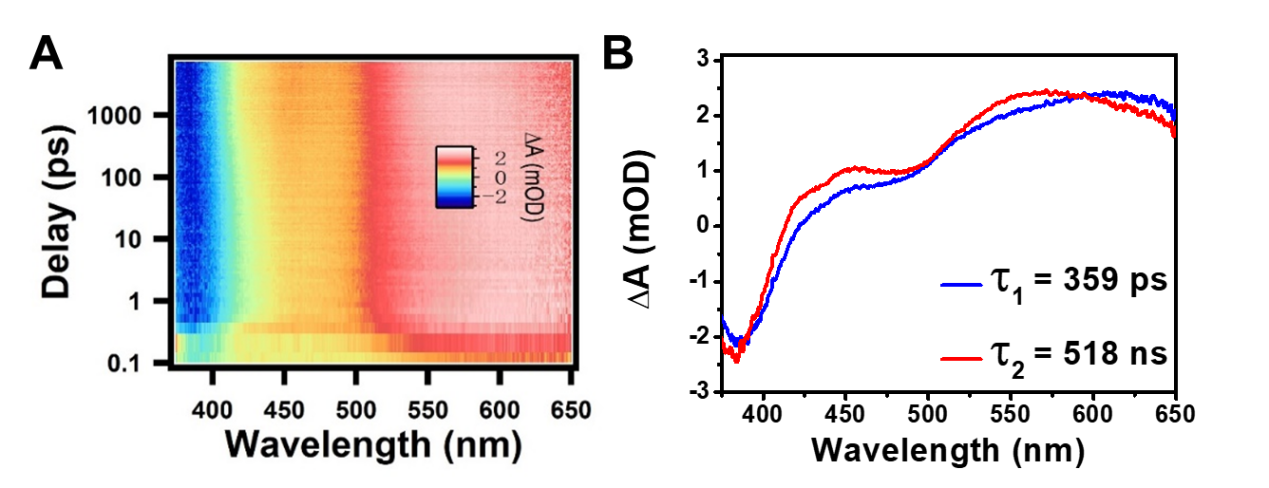


**Figure S35**. (A) TA data map and (B) TA spectra of **Au*_n_*Ag_13-_*_n_*** (20%).


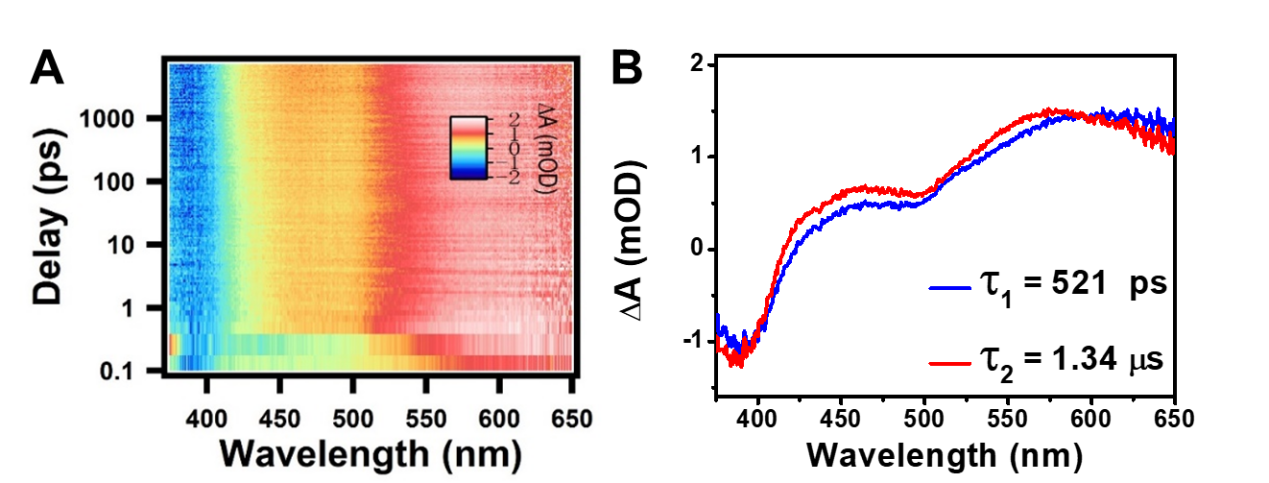


**Figure S36**. (A) TA data map and (B) TA spectra of **Au*_n_*Ag_13-_*_n_*** (30%).

**Figure S37.** The emission spectrum of **Au_13_@PLGA**.

**Figure S38.** The TEM image of **Au_13_**.

**Table S1.** Au, Ag Content of **Au*_n_*Ag_13-_*_n_*** (10%, 20%, and 30%).

|  | **10%** | **20%** | **30%** |
| --- | --- | --- | --- |
| **Ag (Wt.%)** | 26.7812 | 255389 | 24.6296 |
| **Au (Wt.%)** | 3.2153 | 4.2636 | 6.5189 |

**Table S2.** Quantum Yields of **Ag_13_**, **Au*_n_*Ag_13_*_-n_*** (10%, 20%, and 30%), and **Au_13_** in DMSO Solution.

|  | **Ag_13_** | **10%** | **20%** | **30%** | **Au_13_** |
| --- | --- | --- | --- | --- | --- |
| **DMSO** | 0 | 2.5% | 14.1% | 21.5% | 45% |
| **Solid** | 1.5% | 9.1% | 46.2% | 38.5% | 20% |

**Table S3.** Calculated and Experimental Vertical Emission Energy of **Ag_13_**, **Au_1_Ag_12_**, **Au_3_Ag_10_**, **and Au_13_**.

|  | **Exp-*E*_em_**  **eV** | S_1_-S_0_ | | T_1_-S_0_ | | T_2_-S_0_ | |
| --- | --- | --- | --- | --- | --- | --- | --- |
|  |  | **Cal-*E*_em_**  **eV** | **Major orbital contributions** | **Cal-*E*_em_**  **eV** | **Major orbital contributions** | **Cal-*E*_em_**  **eV** | **Major orbital contributions** |
| **Ag_13_** | 1.65 | 1.84 | H→L  98% | 1.75 | H→L  98.5% | / | / |
| **Au_1_Ag_12_** | 1.92 | 2.02 | H→L  97.9% | 2.16 | H→L  88.7% | / | / |
| **Au_3_Ag_10_** | 1.77 | 2.10 | H→L  97.4% | 1.77 | H→L  97.9% | / | / |
| **Au_13_** | 1.56 | 1.35 | H→L  99.2% | 1.27 | H→L  99.1% | 1.77 | H-1→L  96.3% |

**Table S4.** The Spin-orbit Coupling Matrix Elements ($S_{n}\left| Ĥsoc \right|T_{m}$, cm^-1^) Values of **Au*_n_*Ag_13-_*_n_* (*n* = 0, 1, 2, 3, 13)**.

| **Ag_13_** | | **Au_1_Ag_12_** | | **Au_3_Ag_10_** | | **Au_13_** | |
| --- | --- | --- | --- | --- | --- | --- | --- |
| $\left\langle T_{1}\left\vertĤsoc \right\vert S_{0} \right\rangle$  / | 16.06  / | $\left\langle T_{1}\left\vertĤsoc \right\vert S_{0} \right\rangle$  / | 40.49  / | $\left\langle T_{1}\left\vertĤsoc \right\vert S_{0} \right\rangle$  / | 213.50  / | $\left\langle T_{1}\left\vertĤsoc \right\vert S_{0} \right\rangle$  $\left\langle T_{2}\left\vertĤsoc \right\vert S_{0} \right\rangle$ | 98.94  124.16 |
| $\left\langle S_{1}\left\vertĤsoc \right\vert T_{1} \right\rangle$ | 8.83 | $\left\langle S_{1}\left\vertĤsoc \right\vert T_{1} \right\rangle$ | 23.97 | $\left\langle S_{1}\left\vertĤsoc \right\vert T_{1} \right\rangle$ | 45.55 | $\left\langle S_{1}\left\vertĤsoc \right\vert T_{1} \right\rangle$ | 31.62 |

**Table S5.** The Spin-orbit Coupling Matrix Elements ($S_{2}\left| Ĥsoc \right|T_{m}$, cm^-1^) Values of **Au_13_**.

| **Au_13_** | |
| --- | --- |
| $\left\langle S_{2}\left\vertĤsoc \right\vert T_{4} \right\rangle$ | 33.49 |
| $\left\langle S_{2}\left\vertĤsoc \right\vert T_{3} \right\rangle$ | 85.73 |
| $\left\langle S_{2}\left\vertĤsoc \right\vert T_{2} \right\rangle$ | 89.75 |

**Table S6.** S_0_, S_1_, and T_1_ Geometries Bond Length of **Ag_13_** (It only shows that the change of bond length is greater than 0.05 Å).

|  | **S_0_（Å）** | **S_1_（Å）** | **T_1_（Å）** |  | **S_0_（Å）** | **S_1_（Å）** | **T_1_（Å）** |
| --- | --- | --- | --- | --- | --- | --- | --- |
| **Ag13-Ag11** | 2.934 | 2.984 | 2.949 | **Ag1-Ag11** | 3.015 | 3.127 | 3.237 |
| **Ag13-Ag3** | 2.935 | 2.842 | 2.865 | **Ag1-Ag7** | 2.964 | 3.066 | 3.050 |
| **Ag9-Ag2** | 2.921 | 2.895 | 2.878 | **Ag1-Ag10** | 2.952 | 3.013 | 3.010 |
| **Ag9-Ag1** | 2.960 | 2.858 | 2.889 | **Ag11-Ag4** | 2.987 | 2.835 | 2.817 |
| **Ag9-Ag6** | 2.977 | 3.096 | 3.054 | **Ag11-Ag10** | 2.966 | 3.068 | 3.087 |
| **Ag5-Ag7** | 2.830 | 2.879 | 2.882 | **Ag11-Ag12** | 2.964 | 2.905 | 2.911 |
| **Ag5-Ag11** | 2.834 | 2.920 | 2.906 | **Ag4-Ag3** | 2.988 | 3.100 | 3.105 |
| **Ag5-Ag6** | 2.834 | 2.920 | 2.906 | **Ag4-Ag12** | 2.983 | 2.905 | 2.940 |
| **Ag5-Ag4** | 2.840 | 2.879 | 2.896 | **Ag6-Ag5** | 2.980 | 2.832 | 2.820 |
| **Ag2-Ag4** | 2.969 | 3.076 | 3.074 | **Ag6-Ag8** | 2.999 | 2.904 | 2.922 |
| **Ag2-Ag6** | 2.974 | 3.102 | 3.154 | **Ag6-Ag3** | 2.956 | 3.075 | 3.078 |
| **Ag2-Ag3** | 3.022 | 3.127 | 3.139 | **Ag7-Ag10** | 3.009 | 3.173 | 3.191 |
| **Ag2-Ag12** | 2.992 | 3.020 | 2.970 | **Ag8-Ag3** | 3.006 | 2.957 | 2.977 |

**Table S7.** S_0_, S_1_, and T_1_ Geometries Bond Length Change of **Au_1_Ag_12_** (It only shows that the change of bond length is greater than 0.05 Å).

|  | **S_0_（Å）** | **S_1_（Å）** | **T_1_（Å）** |  | **S_0_（Å）** | **S_1_（Å）** | **T_1_（Å）** |
| --- | --- | --- | --- | --- | --- | --- | --- |
| **Ag12-Ag6** | 2.977 | 3.013 | 2.914 | **Ag8-Ag5** | 2.991 | 3.071 | 3.041 |
| **Ag12-Ag11** | 2.957 | 3.021 | 2.989 | **Ag8-Ag10** | 2.968 | 2.942 | 2.912 |
| **Ag12-Ag9** | 2.976 | 2.918 | 2.894 | **Ag3-Ag10** | 2.977 | 2.970 | 3.020 |
| **Ag12-Ag2** | 2.943 | 2.997 | 3.020 | **Ag3-Ag6** | 3.024 | 3.005 | 3.186 |
| **Ag11-Ag3** | 2.955 | 2.902 | 2.899 | **Ag3-Ag1** | 2.984 | 3.030 | 2.939 |
| **Ag11-Ag1** | 2.982 | 2.950 | 3.035 | **Ag6-Ag11** | 2.972 | 3.031 | 3.024 |
| **Ag11-Ag10** | 2.970 | 2.902 | 2.952 | **Ag6-Ag10** | 2.958 | 2.976 | 3.030 |
| **Ag11-Ag8** | 2.964 | 3.052 | 2.972 | **Ag4-Ag5** | 3.036 | 3.062 | 3.117 |
| **Ag11-Ag5** | 2.927 | 3.007 | 2.907 | **Ag4-Ag9** | 2.990 | 3.033 | 3.091 |
| **Au1-Ag3** | 2.839 | 2.922 | 2.840 | **Ag4-Ag11** | 3.016 | 2.987 | 2.942 |
| **Au1-Ag4** | 2.842 | 2.950 | 3.035 | **Ag1-Ag11** | 3.013 | 2.914 | 2.985 |
| **Au1-Ag2** | 2.844 | 2.853 | 2.922 | **Ag1-Ag5** | 2.986 | 3.039 | 2.990 |

**Table S8.** S_0_, S_1_, and T_1_ Geometries Bond Length Change **Au_3_Ag_10_** (It only shows that the change of bond length is greater than 0.05 Å).

|  | **S_0_（Å）** | **S_1_（Å）** | **T_1_（Å）** |  | **S_0_（Å）** | **S_1_（Å）** | **T_1_（Å）** |
| --- | --- | --- | --- | --- | --- | --- | --- |
| **Ag10-Ag9** | 2.978 | 3.029 | 2.929 | **Ag3-Au3** | 2.974 | 2.987 | 2.811 |
| **Ag10-Ag8** | 3.001 | 2.873 | 2.956 | **Ag4-Ag7** | 2.978 | 3.112 | 3.003 |
| **Ag6-Ag3** | 2.976 | 2.886 | 2.979 | **Ag4-Ag8** | 2.978 | 3.113 | 2.976 |
| **Ag6-Ag4** | 2.912 | 2.960 | 2.929 | **Ag7-Ag8** | 2.983 | 3.056 | 3.151 |
| **Ag1-Ag3** | 2.983 | 2.984 | 3.087 | **Ag7-Au3** | 2.966 | 2.905 | 3.107 |
| **Ag1-Ag9** | 3.037 | 3.014 | 3.113 | **Au3-Ag2** | 3.049 | 3.047 | 3.274 |
| **Ag1-Au2** | 2.964 | 2.966 | 3.088 | **Ag2-Ag5** | 2.994 | 2.936 | 2.982 |
| **Au1-Au2** | 2.808 | 2.823 | 2.891 | **Ag2-Ag8** | 2.987 | 3.014 | 3.062 |
| **Au1-Au3** | 2.806 | 2.843 | 2.925 | **Ag8-Au2** | 2.988 | 3.041 | 2.827 |
| **Au2-Ag2** | 2.871 | 2.963 | 2.880 | **Au2-Ag9** | 3.012 | 2.948 | 3.171 |
| **Ag3-Ag9** | 2.963 | 3.047 | 3.031 | **Ag9-Ag5** | 2.972 | 3.070 | 2.961 |
| **Ag3-Ag5** | 3.047 | 3.159 | 2.986 | **/** | **/** | **/** | **/** |

**Table** **S9**. S_0_, S_1_, T_1_, and T_2_ geometries bond length **Au_13_** (It only shows that the change of bond length is greater than 0.05 Å).

|  | **S_0_（Å）** | **S_2_（Å）** | **T_1_（Å）** | **T_2_（Å）** |  | **S_0_（Å）** | **S_2_（Å）** | **T_1_（Å）** | **T_2_（Å）** |
| --- | --- | --- | --- | --- | --- | --- | --- | --- | --- |
| **Au4-Au9** | 2.952 | 2.859 | 2.870 | 2.872 | **Au5-Au9** | 3.000 | 3.168 | 3.207 | 3.196 |
| **Au4-Au3** | 2.973 | 2.895 | 2.895 | 2892 | **Au9-Au10** | 3.171 | 3.390 | 3.416 | 3.399 |
| **Au13-Au10** | 2.948 | 2.884 | 2.886 | 2.887 | **Au9-Au13** | 3.009 | 3.143 | 3.115 | 3.105 |
| **Au13-Au7** | 2.967 | 2.880 | 2.891 | 2.887 | **Au9-Au8** | 3.008 | 2.954 | 2.944 | 2.940 |
| **Au1-Au12** | 2.878 | 2.996 | 2.972 | 2.969 | **Au8-Au13** | 3.154 | 3.074 | 3.058 | 3.048 |
| **Au1-Au5** | 2.882 | 2.962 | 2.956 | 2.961 | **Au5-Au12** | 3.022 | 3.620 | 3.646 | 3.727 |
| **Au1-Au12** | 2.884 | 3.145 | 3.131 | 3.217 | **Au3-Au7** | 3.008 | 3.041 | 3.062 | 3.052 |
| **Au1-Au13** | 2.901 | 2.983 | 2.965 | 2.987 | **Au12-Au13** | 3.022 | 2.807 | 2.803 | 2.793 |
| **Au2-Au3** | 2.999 | 3.155 | 3.101 | 3.121 | **Au12-Au7** | 3.008 | 3.232 | 3.235 | 3.345 |
| **Au2-Au7** | 3.160 | 3.537 | 3.497 | 3.479 | **Au6-Au10** | 3.027 | 2.957 | 2.967 | 2.964 |
| **Au5-Au10** | 2.995 | 3.157 | 3.171 | 3.190 | **Au13-Au10** | 3.034 | 3.196 | 3.187 | 3.182 |

**Table** **S10**. Calculated Basis Function Contribution to Hole and Electron of S_1_, S_2_, and T_1_ for the S_1_, S_2_, and T_1_ Geometries of **Ag_13_**, respectively.

|  | **Atom** | **Shell** | **Type** | **Hole** | **Atom** | **Shell** | **Type** | **Electron** |
| --- | --- | --- | --- | --- | --- | --- | --- | --- |
| **S_1_** | 8(Ag) | 59 | s | 29.59% | 12(Ag) | 93 | p_z_ | 6.13% |
|  | 2(Ag) | 11 | s | 28.53% | 10(Ag) | 77 | p_z_ | 6.1% |
|  | 5(Ag) | 37 | p_x_ | 15.95% | 7(Ag) | 53 | p_x_ | 5.93% |
|  | 1(Ag) | 3 | s | 11.06% | 4(Ag) | 29 | p_x_ | 5.27% |
|  | 11(Ag) | 83 | s | 10.41% | 3(Ag) | 17 | s | 4.81% |
|  | 6(Ag) | 43 | s | 7.79% | 6(Ag) | 41 | s | 4.3% |
|  | 8(Ag) | 57 | s | 6.63 | 7(Ag) | 49 | s | 3.61% |
|  | 3(Ag) | 19 | s | 6.2% | 6(Ag) | 45 | p_x_ | 3.1% |
|  | 4(Ag) | 27 | s | 6.18% | 3(Ag) | 19 | s | 2.99% |
|  | 2(Ag) | 9 | s | 6.09% | 7(Ag) | 51 | s | 2.95% |
| **S_2_** | 10(Ag) | 75 | s | 27.97% | 4(Ag) | 29 | p_y_ | 6.32% |
|  | 12(Ag) | 91 | s | 26.71% | 11(Ag) | 85 | p_y_ | 6.01% |
|  | 13(Ag) | 99 | s | 10.11% | 6(Ag) | 43 | s | 5.57% |
|  | 9(Ag) | 67 | s | 9.96% | 7(Ag) | 53 | p_y_ | 5.07% |
|  | 2(Ag) | 11 | s | 9.61% | 3(Ag) | 17 | s | 5.04% |
|  | 8(Ag) | 59 | s | 9.25% | 6(Ag) | 41 | s | 4.61% |
|  | 5(Ag) | 37 | p_z_ | 8.53% | 1(Ag) | 5 | p_y_ | 4.18% |
|  | 5(Ag) | 37 | p_y_ | 7.05% | 4(Ag) | 27 | s | 3.97% |
|  | 10(Ag) | 73 | s | 6.33% | 4(Ag) | 25 | s | 3.02% |
|  | 12(Ag) | 89 | s | 5.97% | 1(Ag) | 5 | p_x_ | 2.68% |
| **T_1_** | 8(Ag) | 59 | s | 26.07% | 4(Ag) | 29 | p_x_ | 5.9 |
|  | 2(Ag) | 11 | s | 24.31% | 12(Ag) | 93 | p_z_ | 5.69 |
|  | 5(Ag) | 37 | p_x_ | 19% | 7(Ag) | 53 | p_x_ | 5.56 |
|  | 1(Ag) | 3 | s | 15.8% | 10(Ag) | 77 | p_z_ | 5.18 |
|  | 11(Ag) | 83 | s | 15.21% | 3(Ag) | 17 | s | 5.09 |
|  | 6(Ag) | 43 | s | 9.85% | 3(Ag) | 21 | p_x_ | 4.87 |
|  | 3(Ag) | 19 | s | 7.73% | 6(Ag) | 41 | s | 4.38 |
|  | 8(Ag) | 57 | s | 6.21% | 6(Ag) | 45 | p_x_ | 4.21 |
|  | 2(Ag) | 9 | s | 5.38% | 7(Ag) | 49 | s | 3.7 |
|  | 4(Ag) | 27 | s | 5.1% | 9(Ag) | 69 | p_y_ | 3.21 |

**Table** **S11**. Calculated Basis Function Contribution to Hole and Electron of S_1_, and T_1_ for the S_1_, and T_1_ Geometries of **Au_1_Ag_12_**, respectively.

|  | **Atom** | **Shell** | **Type** | **Hole** | **Atom** | **Shell** | **Type** | **Electron** |
| --- | --- | --- | --- | --- | --- | --- | --- | --- |
| **S_1_** | 6(Ag) | 43 | s | 12.6% | 28(N) | 215 | p_y_ | 9.07% |
|  | 7(Ag) | 51 | s | 11.91% | 186(C) | 887 | p_y_ | 8.28% |
|  | 1(Au) | 5 | p_x_ | 7.99% | 182(C) | 871 | p_y_ | 7.72% |
|  | 6(Ag) | 41 | s | 5.98% | 182(C) | 873 | p_y_ | 6.14% |
|  | 12(Ag) | 91 | s | 5.67% | 186(C) | 889 | p_y_ | 5.9% |
|  | 1(Au) | 5 | p_z_ | 5.51% | 28(N) | 217 | p_y_ | 5.38% |
|  | 9(Ag) | 67 | s | 4.56% | 180(C) | 863 | p_y_ | 4.08% |
|  | 7(Ag) | 49 | s | 4.43% | 4(Ag) | 29 | p_y_ | 3.5% |
|  | 21(P) | 165 | p_x_ | 3.58% | 180(C) | 865 | p_y_ | 3.26% |
|  | 7(Ag) | 53 | p_x_ | 3.46% | 182(C) | 871 | p_z_ | 2.76% |
| **T_1_** | 1(Au) | 5 | p_y_ | 14.6% | 231(C) | 1077 | p_y_ | 7.38% |
|  | 7(Ag) | 51 | s | 12.6% | 29(N) | 221 | p_y_ | 6.3% |
|  | 6(Ag) | 43 | s | 12.32% | 234(C) | 1091 | p_y_ | 5.27% |
|  | 10(Ag) | 75 | s | 6.77% | 231(C) | 1079 | p_y_ | 5.18% |
|  | 11(Ag) | 83 | s | 6.15% | 236(C) | 1099 | p_y_ | 4.6% |
|  | 4(Ag) | 27 | s | 5.87% | 3(Ag) | 21 | p_y_ | 4.15% |
|  | 5(Ag) | 35 | s | 5.76% | 234(C) | 1093 | p_y_ | 4.1% |
|  | 7(Ag) | 49 | s | 5.3% | 29(N) | 223 | p_y_ | 3.88% |
|  | 6(Ag) | 41 | s | 5.12% | 236(C) | 1101 | p_y_ | 3.73% |
|  | 7(Ag) | 53 | p_y_ | 2.66% | 231(C) | 1077 | p_z_ | 2.34% |

**Table** **S12**. Calculated Basis Function Contribution to Hole and Electron of S_1_, and T_1_ for the S_1_, and T_1_ Geometries of **Au_3_Ag_10_**, respectively.

|  | **Atom** | **Shell** | **Type** | **Hole** | **Atom** | **Shell** | **Type** | **Electron** |
| --- | --- | --- | --- | --- | --- | --- | --- | --- |
| **S_1_** | 1(Au) | 5 | p_x_ | 11.52% | 231(C) | 1077 | p_x_ | 9.74% |
|  | 6(Ag) | 43 | s | 10.46% | 29(N) | 221 | p_x_ | 7.96% |
|  | 12(Ag) | 91 | s | 9.73% | 231(C) | 1079 | p_x_ | 6.97% |
|  | 7(Ag) | 51 | s | 9.46% | 236(C) | 1099 | p_x_ | 6.28% |
|  | 9(Ag) | 67 | s | 8.54% | 234(C) | 1091 | p_x_ | 5.94% |
|  | 10(Ag) | 75 | s | 5.58% | 236(C) | 1101 | p_x_ | 5.15% |
|  | 4(Ag) | 27 | s | 5.2% | 234(C) | 1093 | p_x_ | 4.74% |
|  | 6(Ag) | 41 | s | 4.8% | 29(N) | 223 | p_x_ | 4.49% |
|  | 7(Ag) | 49 | s | 4.08% | 231(C) | 1077 | p_z_ | 2.91% |
|  | 12(Ag) | 89 | s | 3.93% | 236(C) | 1099 | p_z_ | 2.71% |
| **T_1_** | 1(Au) | 5 | p_x_ | 14.29% | 11(Au) | 83 | s | 7.46% |
|  | 9(Ag) | 67 | s | 6.88% | 5(Au) | 35 | s | 5.91% |
|  | 3(Ag) | 19 | s | 6.81% | 4(Ag) | 29 | p_x_ | 5.3% |
|  | 12(Ag) | 91 | s | 5.84% | 11(Au) | 85 | p_x_ | 5.22% |
|  | 2(Ag) | 11 | s | 5.69% | 10(Ag) | 77 | p_x_ | 4.45% |
|  | 4(Ag) | 27 | s | 5.42% | 5(Au) | 37 | p_x_ | 4.25% |
|  | 3(Ag) | 17 | s | 5.4% | 1(Au) | 7 | d_yz_ | 3.31% |
|  | 10(Ag) | 75 | s | 4.76% | 13(Ag) | 101 | p_y_ | 3.2% |
|  | 9(Ag) | 65 | s | 4.73% | 7(Ag) | 53 | p_z_ | 3.17% |
|  | 12(Ag) | 89 | s | 4.28% | 10(Ag) | 75 | s | 2.76% |

**Table S13**. Calculated Basis Function Contribution to Hole and Electron of S_1_, S_2_, T_1_, and T_2_ for the S_1_, S_2_, T_1_, and T_2_ Geometries of **Au_13_**, respectively.

|  | **Atom** | **Shell** | **Type** | **Hole** | **Atom** | **Shell** | **Type** | **Electron** |
| --- | --- | --- | --- | --- | --- | --- | --- | --- |
| **S_1_** | 1(Au) | 6 | p_x_ | 34.19% | 12(Au) | 91 | s | 6.58% |
|  | 1(Au) | 5 | p_x_ | 13.41% | 5(Au) | 35 | s | 5.24% |
|  | 11(Au) | 87 | d_xz_ | 4.06% | 2(Au) | 11 | s | 5.11% |
|  | 4(Au) | 31 | d_xz_ | 3.91% | 13(Au) | 101 | p_x_ | 4.34% |
|  | 1(Au) | 6 | p_y_ | 2.78% | 4(Au) | 29 | p_y_ | 3.51% |
|  | 2(Au) | 15 | d_xy_ | 1.77% | 8(Au) | 61 | p_z_ | 3.34% |
|  | 19(P) | 149 | p_x_ | 1.74% | 1(Au) | 7 | d_yz_ | 3.25% |
|  | 18(P) | 141 | p_x_ | 1.55% | 11(Au) | 85 | p_y_ | 3.13% |
|  | 23(P) | 181 | p_x_ | 1.48% | 2(Au) | 13 | p_x_ | 3.06% |
|  | 12(Au) | 95 | d_x_^2^_-y_^2^ | 1.46% | 13(Au) | 99 | s | 2.91% |
| **S_2_** | 1(Au) | 6 | p_y_ | 38.52 | 12(Au) | 91 | s | 6.25% |
|  | 1(Au) | 5 | p_y_ | 7.93 | 5(Au) | 35 | s | 4.92% |
|  | 1(Au) | 6 | p_x_ | 6.81 | 2(Au) | 11 | s | 4.6% |
|  | 11(Au) | 87 | d_yz_ | 5.15 | 13(Au) | 101 | p_x_ | 4.09% |
|  | 4(Au) | 31 | d_yz_ | 4.36 | 13(Au) | 99 | s | 3.22% |
|  | 24(P) | 189 | p_y_ | 2.58 | 4(Au) | 29 | p_y_ | 3.05% |
|  | 1(Au) | 5 | p_x_ | 2.15 | 1(Au) | 7 | d_yz_ | 2.85% |
|  | 17(P) | 133 | p_y_ | 1.79 | 2(Au) | 13 | p_x_ | 2.76% |
|  | 6(Au) | 45 | p_y_ | 1.59 | 8(Au) | 61 | p_z_ | 2.73% |
|  | 8(Au) | 61 | p_y_ | 1.51 | 11(Au) | 85 | p_y_ | 2.7% |
| **T_1_** | 1(Au) | 6 | p_x_ | 37.25% | 12(Au) | 91 | s | 6.8% |
|  | 1(Au) | 5 | p_x_ | 13.74% | 5(Au) | 35 | s | 5.43% |
|  | 11(Au) | 87 | d_xz_ | 3.89% | 13(Au) | 101 | p_x_ | 4.83% |
|  | 4(Au) | 31 | d_xz_ | 3.8% | 2(Au) | 11 | s | 4.51% |
|  | 2(Au) | 15 | d_xy_ | 1.72% | 2(Au) | 13 | p_x_ | 3.44% |
|  | 19(P) | 149 | p_x_ | 1.7% | 4(Au) | 29 | p_y_ | 3.42% |
|  | 18(P) | 141 | p_x_ | 1.6% | 1(Au) | 7 | d_yz_ | 3.35% |
|  | 12(Au) | 95 | d_x_^2^_-y_^2^ | 1.36% | 8(Au) | 61 | p_z_ | 3.19% |
|  | 23(P) | 181 | p_x_ | 1.31% | 11(Au) | 85 | p_y_ | 3% |
|  | 6(Au) | 47 | d_xy_ | 1.27% | 5(Au) | 37 | p_x_ | 2.72% |
| **T_2_** | 1(Au) | 6 | p_y_ | 49.45% | 12(Au) | 91 | s | 6.95% |
|  | 1(Au) | 5 | p_y_ | 9.65% | 5(Au) | 35 | s | 5.6% |
|  | 11(Au) | 87 | d_yz_ | 6.12% | 13(Au) | 101 | p_x_ | 5.1% |
|  | 4(Au) | 31 | d_yz_ | 5.07% | 2(Au) | 11 | s | 4.68% |
|  | 24(P) | 189 | p_y_ | 2.3% | 4(Au) | 29 | p_y_ | 3.22 |
|  | 25(P) | 197 | p_y_ | 2.15% | 1(Au) | 7 | d_yz_ | 3.16% |
|  | 13(Au) | 103 | d_x_^2^-_y_^2^ | 1.75% | 2(Au) | 13 | p_x_ | 3.13% |
|  | 13(Au) | 101 | p_x_ | 1.47% | 11(Au) | 85 | p_y_ | 3.03% |
|  | 1(Au) | 6 | p_x_ | 1.43% | 8(Au) | 61 | p_z_ | 3.02% |
|  | 17(P) | 133 | p_y_ | 1.38% | 6(Au) | 45 | p_z_ | 2.54% |

**Table** **S14**. Crystal Data and Structure Refinements for **Ag_13_**, **Au_1_Ag_12,_** and **Au_13_**.

|  | **Ag_13_** | **Au_1_Ag_12_** | **Au_13_** |
| --- | --- | --- | --- |
| **Empirical formula** | C_145_H_115_Ag_13_Cl_2_F_18_N_5_P_10_Sb_3_ | C1_46.5_H_118_Ag_12_AuCl_5_F_18_N_5_P_10_Sb_3_ | C_145_H_115_Au_13_Cl_2_F_18_N_5_P_10_Sb_3_ |
| **Formula weight** | 4417.57 | 4634.06 | 5575.83 |
| **Temperature/K** | 200.00(10) | 200.00(10) | 200.00(10) |
| **Crystal system** | triclinic | triclinic | triclinic |
| **Space group** | *P*-1 | *P*-1 | *P*-1 |
| **a/Å** | 19.2451(3) | 19.15370(10) | 22.35980(10) |
| **b/Å** | 20.2312(3) | 20.22730(10) | 24.1935(2) |
| **c/Å** | 22.8333(4) | 22.82880(10) | 34.4724(3) |
| **α/°** | 97.1830(10) | 97.2330(10) | 71.8180(10) |
| **β/°** | 99.5450(10) | 99.4810(10) | 79.0210(10) |
| **γ/°** | 109.6670(10) | 109.4580(10) | 88.1620(10) |
| **Volume/Å3** | 8097.6(2) | 8068.55(9) | 17385.4(2) |
| **Z** | 2 | 2 | 4 |
| **ρcalcg/cm3** | 1.812 | 1.907 | 2.13 |
| **μ/mm‒1** | 17.989 | 19.232 | 25.341 |
| **F(000)** | 4260 | 4450 | 10184 |
| **Crystal size/mm3** | 0.106 × 0.05 × 0.04 | 0.06 × 0.04 × 0.03 | 0.05 × 0.03 × 0.02 |
| **Radiation** | CuKα (λ = 1.54184) | CuKα (λ = 1.54184) | Cu Kα (λ = 1.54184 Å) |
| **2θrange for data collection/°** | 5.488 to 147.694 | 5.684 to 147.442 | 3.968 to 147.71 |
| **Index ranges** | -23 ≤ h ≤ 23, -25 ≤ k ≤ 24, -28 ≤ l ≤ 28 | -23 ≤ h ≤ 23, -25 ≤ k ≤ 18, -28 ≤ l ≤ 28 | -26 ≤ h ≤ 21, -30 ≤ k ≤ 29, -42 ≤ l ≤ 33 |
| **Reflections collected** | 31694 | 31503 | 186153 |
| **Independent reflections** | 31694 [Rint = 0.0673, Rsigma = 0.0657] | 31503 [Rint = 0.0406, Rsigma = 0.0457] | 68048 [Rint = 0.0768, Rsigma = 0.0787] |
| **Data/restraints/parameters** | 31694/623/1902 | 31503/115/1856 | 68048/3326/3752 |
| **Goodness-of-fit on F2** | 1.089 | 1.021 | 1.052 |
| **Final R indexes [I>=2σ (I)]** | R1 = 0.0947, wR2 = 0.2388 | R1 = 0.0497, wR2 = 0.1212 | R1 = 0.0808, wR2 = 0.2237 |
| **Final R indexes [all data]** | R1 = 0.1088, wR2 = 0.2447 | R1 = 0.0602, wR2 = 0.1271 | R1 = 0.0947, wR2 = 0.2395 |
| **Largest diff. peak/hole / e Å-3** | 3.00/-1.97 | 1.68/-2.22 | 6.28/-3.99 |
| **CCDC number** | 2320418 | 2320435 | 2320428 |

*R*_1_ = ∑׀׀*F*_o_׀−׀*F*_c_׀׀/∑׀*F*_o_׀. *wR*_2_ = [∑*w*(*F*_o_^2^ −*F*_c_^2^)^2^/∑*w*(*F*_o_^2^)^2^]^1/2^
